# Proteomics-based characterization of ribosome heterogeneity in adult mouse organs

**DOI:** 10.1007/s00018-025-05708-7

**Published:** 2025-04-24

**Authors:** Marie R. Brunchault, Anne-Marie Hesse, Julia Schaeffer, Albrecht Fröhlich, Ana Saintpierre, Charlotte Decourt, Florence Combes, Homaira Nawabi, Yohann Couté, Stephane Belin

**Affiliations:** 1 Univ. Grenoble Alpes, Inserm, U1216, Grenoble Institut Neurosciences, 38000 Grenoble, France; 2 Present Address: IBDM, CNRS, UMR 7288, Aix-Marseille Université, Marseille, France; 3 Present Address: Univ. Grenoble Alpes, INSERM, CEA, UA13 BGE, CNRS, CEA, FR2048, 38000 Grenoble, France

**Keywords:** Ribosome, Ribosome heterogeneity, Mass spectrometry, Quantitative proteomic, Translational control, Transcriptomic analysis

## Abstract

The translation process, leading to protein synthesis from mRNA, has been long thought to be invariable in all cellular organisms. Increasing evidence shows that it is finely regulated by variable features of the translation machinery. Notably, ribosomes, the functional units of protein synthesis, are suggested to display variations in their composition, depending on the developmental stage, cell type or physio-pathological context, thus hinting a new level of actionable regulation of gene expression. Yet, a comprehensive map of the heterogeneity of ribosome composition in ribosomal proteins (RPs) in different organs and tissues is not available. In this work, we explored tissue-specific ribosome heterogeneity using mass spectrometry-based quantitative proteomic characterization of ribosomal fractions purified from 14 adult mouse organs and tissues. We performed crossed clustering and statistical analyses of RP composition to highlight stable, variable and tissue-specific RPs across organs and tissues. Focusing on specific RPs, we validated their varying abundances using a targeted proteomic approach and western blot analyses, providing further insights into the tissue-specific ribosome RP signature. Finally, we investigated the origin of RP variations in ribosome fraction of the different tissues, by comparing RP relative amounts in our ribosomal proteomic dataset with their corresponding transcript abundances in three independent transcriptomic datasets. Interestingly, we found that, in some tissues, the RP abundance in purified ribosomes does not always correlate with the corresponding RP transcript level, arguing for a translational regulation of RP expression, and/or a regulated incorporation of RPs into ribosomes. Altogether, our data support the notion of a tissue-specific RP signature of ribosomes, which opens avenues to study how specific ribosomal composition provides an additional level of regulation to control gene expression in different tissues and organs.

**Graphical abstract:**

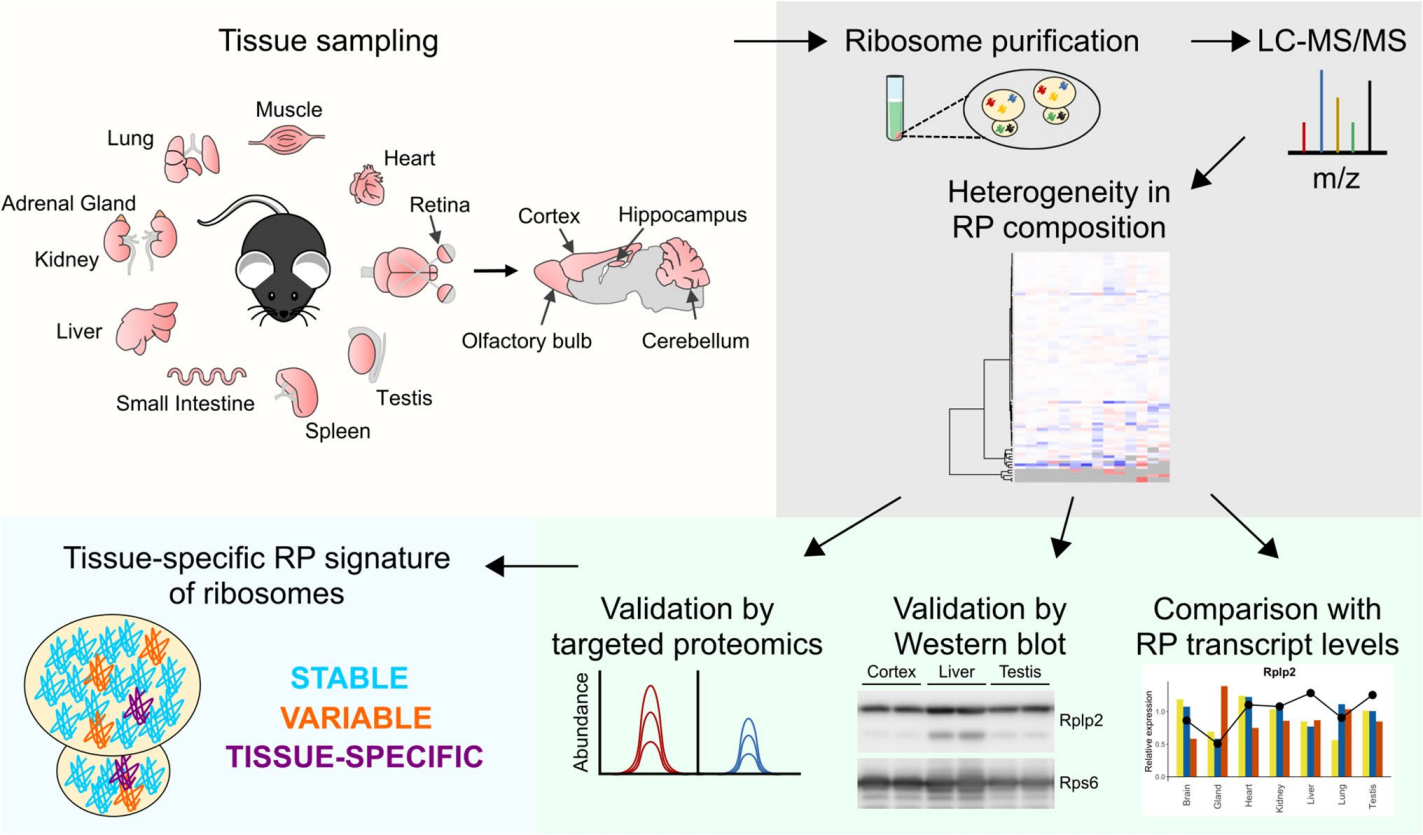

**Supplementary Information:**

The online version contains supplementary material available at 10.1007/s00018-025-05708-7.

## Introduction

A major challenge in cell biology is to understand how genes are expressed and regulated in space and time to control cell identity and specificity, leading to organism homeostasis. Genetic expression is defined by the unidirectional flow “DNA-mRNA-Protein”: after the DNA is transcribed in the nucleus, mRNAs are processed and exported to the cytoplasm to be translated into proteins. Tremendous amount of work has successfully focused on the regulation of the first step of the genetic flow (transcription of DNA into mRNA). The contribution of transcription factors recruited onto promoter and enhancer regions, chromatin accessibility to the transcriptional machinery [1, 2] and epigenetic regulations [3] have been largely documented. Notably, mechanisms regulating gene transcription under normal and pathological conditions have been thoroughly described, as for example during regeneration of the nervous system [4, 5] or in different human diseases [6, 7]. The rapid development and increasing depth of analysis of transcriptomic studies, thanks to next-generation sequencing, accelerate the understanding of the different levels of regulation of mRNA expression and processing.

In many studies, often protein expression levels have been extrapolated from mRNA amounts [8, 9]. Surprisingly, combined analyses of transcriptomes and proteomes in various physio-pathological conditions revealed that mRNA and protein levels generally show only a very partial correlation [10–14]. These results shed light on the critical role of post-transcriptional and translational regulations in the control of genetic expression. Notably, regulation of the translational process is at play to control developmental programs. Alterations in its key regulators can have pathological consequences [15, 16]. Moreover, the translation of alternative proteins deriving from the same mRNA sequence (undetectable by RNA analysis) reinforces the notion that translational control is a major player in the regulation of gene expression [17, 18].

Protein synthesis is a fundamental, high energy-consuming process in cellular life. The ribosome is the main functional unit in this reaction, decoding the information carried by mRNAs to assemble individual amino acids into proteins. It is a large complex composed of ribosomal RNAs (rRNAs) and ribosomal proteins. Even if its overall structure and function have been well-conserved during evolution [19], some differences are observed between organisms. For example, the eukaryotic ribosome has a higher number of ribosomal proteins (RPs) and its rRNAs are longer compared to their prokaryotic counterparts [20]. The mammalian ribosome is composed of 80 RPs and 4 rRNAs, distributed into two subunits: the large 60S subunit contains 46 ribosomal proteins (RPLs) and 3 rRNAs (28S, 5.8S and 5S), while the small 40S subunit is formed by 34 ribosomal proteins (RPSs) and the 18S rRNA. mRNA translation occurs through a tightly ordered sequence of three steps: initiation, elongation and termination. Each step involves specific factors (initiation, elongation and release factors—eIF, eEF and eRF) and aminoacyl-tRNAs. Moreover, the ribosome itself catalyzes peptide bound formation in the newly synthetized proteins [21].

Historically, the common dogma described the ribosome as a stable complex producing proteins from supplied mRNAs, with no regulatory role regardless of the physio-pathological conditions. Surprisingly, recent detailed studies of the translation process have revealed that the ribosome can play a more important role in the regulation of protein expression than initially thought [22, 23]. Indeed, it is suggested that variations in the molecular composition of this complex (rRNA, RPs, translation factors) will influence directly the quantity and/or the quality of translation.

Alterations of ribosome composition linked to specific defects in RPs have been identified in pathologies grouped under the term of “ribosomopathies”. Indeed about 20 different mutated RPs are linked to the Diamond Blackfan anemia, malformations and cancer predisposition [24, 25]. Indeed, somatic point mutations in individual ribosomal proteins impact translational regulation and subsequent protein expression of oncogenic factors, leading to cancer cell proliferation [26, 27]. It also appears that the precise dosage of RPs contributes to maintain homeostasis and their imbalance could lead to pathologies such as the 5q-syndrome, a specific myelodysplastic syndrome caused by the loss of one copy of the gene coding for the ribosomal protein Rps14 [28]. Specific RP variants were reported to impair translational fidelity [29, 30]. In addition, expression and phosphorylation of the ribosomal protein Rps6 contribute to central and peripheral nervous system regeneration capacity [31].

In physiological conditions, early transcriptomic studies revealed that RP paralogs could replace “canonical” counterparts in some organs. For example, the ribosomal protein L3-like (Rpl3l) is only expressed in skeletal muscle and controls myotube formation [32]. Likewise, ribosomal protein L10-like (Rpl10l) is specific to the testis and critical for male meiotic transition [33]. In addition, fine regulation of ribosome composition also appears to be involved in the spatial regulation of protein expression: in yeast, specific RP paralogs are required for localized translation of mRNAs, suggesting a ribosomal code [34]. Specific RPs from the large or small ribosome subunits can be involved in the selection of mRNA sub-pools to be translated, as shown for Rpl10a [35], Rpl40 [36], or Rps25 [35]. This may occur through translational regulatory elements located in transcript untranslated regions (UTR). Of example, during mammalian development, ribosomal protein Rpl38 drives specific translation of HOX genes [37, 38]. Recently, Rpl36a expression has been identified to be increased in mesenchymal ribosomes and sufficient to promote acquisition of mesenchymal features in cells[39]. Moreover, the RP content of ribosomes is distinct between monosomes and polysomes [40] and the modular RP composition of ribosomes controls translation fidelity and efficiency [41].

In addition, rRNA modifications were shown to participate to translation regulation. For instance, rRNA is highly modified with more than hundreds of 2’-O methylation and pseudourydylation. Dysregulation of these rRNA modifications contributes to cancer development via alteration of translational fidelity or modification of CAP/IRES dependent translational initiation of specific mRNA [42–45]. In addition, heterogeneity in the expansion segment of the rRNA could also directly control mRNA translation such as HOXa9 mRNA [46], even if it is still debated [47, 48]. Altogether, these studies pioneered the emerging concept of “specialized ribosomes” and provide a strong link between ribosome heterogeneity, translation specificity and specific phenotypic traits.

Despite evidences that one or even several RPs can be modified or exchanged in specific physio-pathological conditions [49], there is still a lack of comprehensive data describing ribosome heterogeneity in different cell types and tissues. Datasets comparing ribosomes composition in different tissues are mostly derived from mRNA expression levels that cannot be used to directly infer the abundances of RPs into functional ribosomes [50, 51]. Recently, Li and colleagues performed a proteomic analysis of 80S monosomes from nine tissues, providing evidence of variability in RP composition between the analyzed tissues [52]. Alternatively, Alkan et al. developed a prediction tool based on Ribo-Seq data-extracted rRNA fragment positioning in the ribosome to highlight differential incorporation of individual RPs among 6 adult and embryonic mouse organs [53].

In our study, we purified the ribosomal fraction from 14 different adult mouse tissues and analyzed their protein composition using mass spectrometry (MS)-based quantitative proteomics. We show that ribosomes exhibit heterogeneity in RP composition among organs, not only in the case of RP paralogs (e.g. in muscle and testis, consistent with previous works based on transcriptomics), but also for several canonical RPs. Finally, we compared our proteomic results with transcriptomic datasets to decipher the origin of such specialization. Altogether, our work emphasizes the tissue-specific modulation of the RP content of ribosomes and opens the way to the study of its role in the regulation of gene expression.

## Results

### Characterization of ribosomal fractions prepared from different adult mouse tissues

**Fig. 1:**
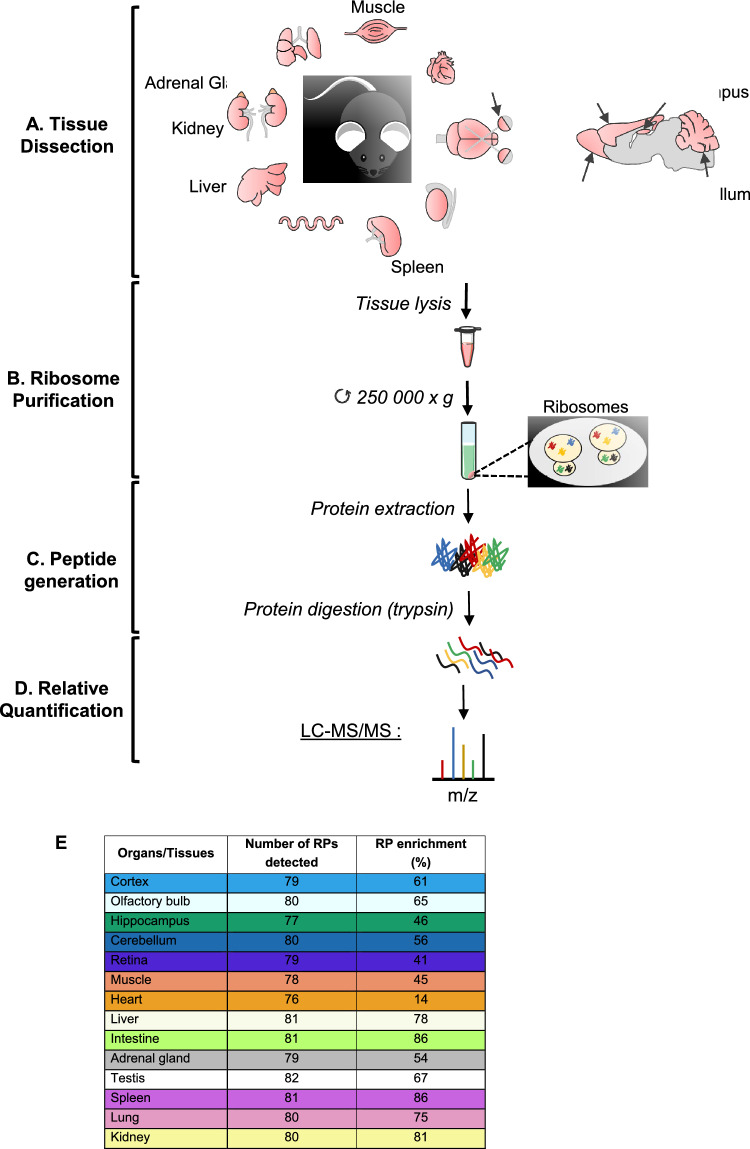
Workflow of MS-based proteomic analysis of ribosomal fractions purified from adult mouse organs. **A** Different wild-type adult mouse tissues were dissected out, lysed and processed for fractionation. Analysis of each tissue or organ has been done using three biological replicates. Created with BioRender.com. **B** After separation of the nuclear and mitochondrial fractions, the ribosome fraction was purified on a sucrose cushion with ultracentrifugation at 250,000 × g for 2 h. **C** Proteins were extracted from the ribosome fraction, loaded on a Bis–Tris polyacrylamide gel and in-gel digested using trypsin. **D** Extracted peptides were analyzed by liquid chromatography coupled to tandem MS for identification and relative quantification of proteins. **E** Summary of the number of RPs reliably detected in at least two replicates and the percentage of RP enrichment for each tissue

To reveal any heterogeneity in RP composition within ribosomes across different tissues, we purified and analyzed the ribosomal fraction of different organs from wild-type (WT) adult (6 week-old) mice (Fig. 1A). 14 different tissues dissected from 11 different organs were studied: lungs, kidneys, adrenal glands, liver, small intestine, spleen, testis, two types of muscle (heart and quadriceps skeletal muscle), various brain regions (cortex, hippocampus, olfactory bulbs, and cerebellum) and retina. For each tissue, we analyzed three independent biological replicates (N = 3 mice, except for small organs for which we pooled tissues from several animals). After tissue lysis, ribosomes were purified by centrifugation through a sucrose cushion as previously described [54] (Fig. 1B). We aimed to obtain the most purified ribosomal fractions, regardless of the purity of other fractions. We confirmed by western blot analysis that the prepared ribosomal fractions were enriched in RPs and contained no or limited contamination using several subcellular fraction markers, e.g. Histone 3 (H3) for the nuclear fraction, Hsp60 for the mitochondrial fraction, GAPDH for the cytoplasmic (post-ribosomal) fraction, and Rps6 and Rpl22 as ribosome components from the small and large subunits (S1A Fig). The efficiency of our ribosome enrichments was also verified by Coomassie blue staining of proteins upon SDS-PAGE separation. For each organ, the profile of the ribosomal fraction was distinct from that of the total fraction and exhibited a strong enrichment in low- to medium-molecular weight proteins that correspond to RPs (11 kDa to 47 kDa in mammals [55]) (S1B Fig).

To further characterize the ribosomal fractions prepared from the different organs and tissues, their protein content was analyzed by nano-liquid chromatography (LC) coupled to MS-based quantitative proteomics (Fig. 1C, D). 85 different RPs (36 RPSs and 49 RPLs) were identified in at least three replicates of one tissue/organ across the 14 analyzed tissues/organs (S1 Table). For each tissue, the number of RPs reliably detected in at least two replicates ranged from 76 to 82 (Fig. 1E). Among them, several paralogs were identified: Rpl10l, Rpl22l1, Rpl39l, Rpl3l, Rpl7l1, Rps27l and Rps4l (S1 Table). As calculated using the intensity-based absolute quantification (iBAQ) metrics [10], RPs accounted for more than 40% of the total protein amount in ribosomal fractions prepared from most tissues, and notably > 80% for intestine, spleen and kidney, indicating a strong enrichment in ribosomes. Only the heart ribosomal fraction presented a lower RP enrichment, reaching only 14% in our different purification attempts (Fig. 1E).

**Fig. 2:**
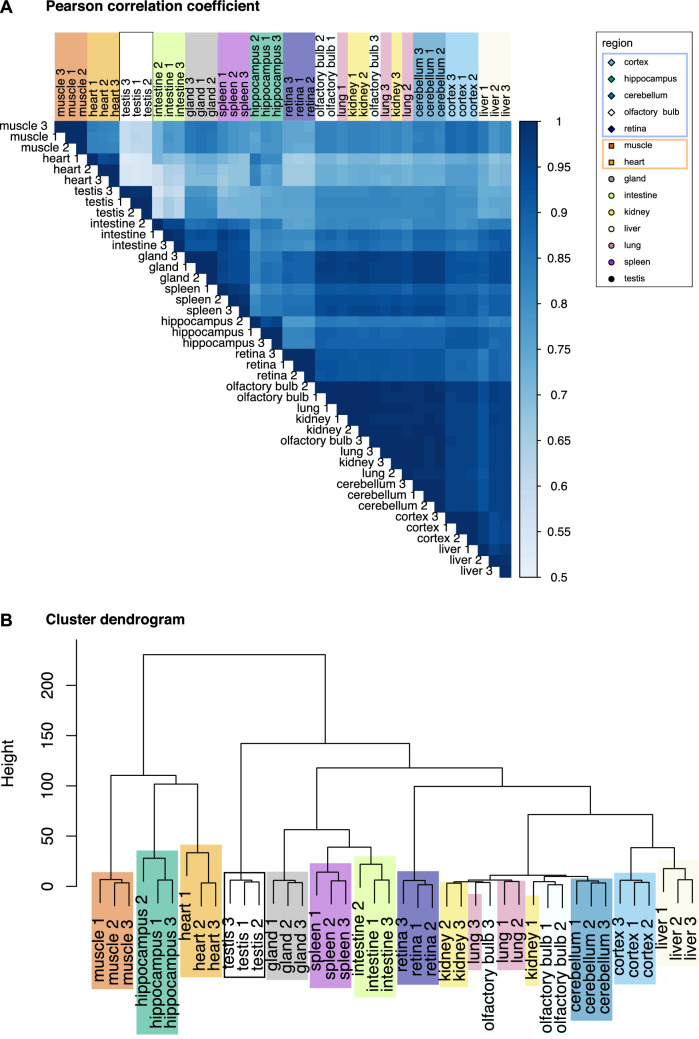
Clustering of ribosomal fractions from adult mouse organs based on RP normalized abundances. **A** Matrix of correlation showing clustering of biological replicates of ribosomal fractions from the 14 adult mouse tissues (Pearson correlation coefficient). **B** Dendrogram showing hierarchical clustering of biological replicates of ribosomal fractions of the 14 adult mouse tissues. Hierarchical clustering is computed from the Euclidian distance between the log-transformed relative abundances of RP, with the Ward’s clustering method

To assess the reproducibility of our workflow, we analyzed the correlation of the measured abundances of the different RPs in the different replicates prepared from each tissue (Fig. 2A, S2 Fig). The correlation coefficients indicate a high consistency in the RP abundances measured in biological replicates for all analyzed tissues. These results further confirmed the efficiency of our purification procedure to reproducibly enrich ribosomes from the different organs and tissues.

### RP composition as a signature of ribosomal fractions specific to adult organs and tissues

We then studied the distribution of individual RPs in the ribosomal fractions enriched from the different tissues. For each RP, we normalized the extracted abundances to the mean abundance of all samples and performed a clustering analysis after log-transformation. The dendrogram representation revealed that the three biological replicates of each tissue generally clustered together better than with the replicates of the other tissues (Fig. 2B). This suggests some heterogeneity in the relative abundance of RPs in ribosomal fractions from different tissues.

This is exemplified by the absence of detection of a few canonical RPs in some tissues, as Rps15 detected in all tissues but the adrenal gland, intestine and muscle, and Rps23 detected in all but hippocampus and heart (S1 Table). This differential detection among organs/tissues was exacerbated for several paralogous RPs that were only detected in a limited number of tissues, e.g. Rpl3l specifically detected in heart and muscle, and Rpl39l and Rpl10l specifically detected in testis.

**Fig. 3:**
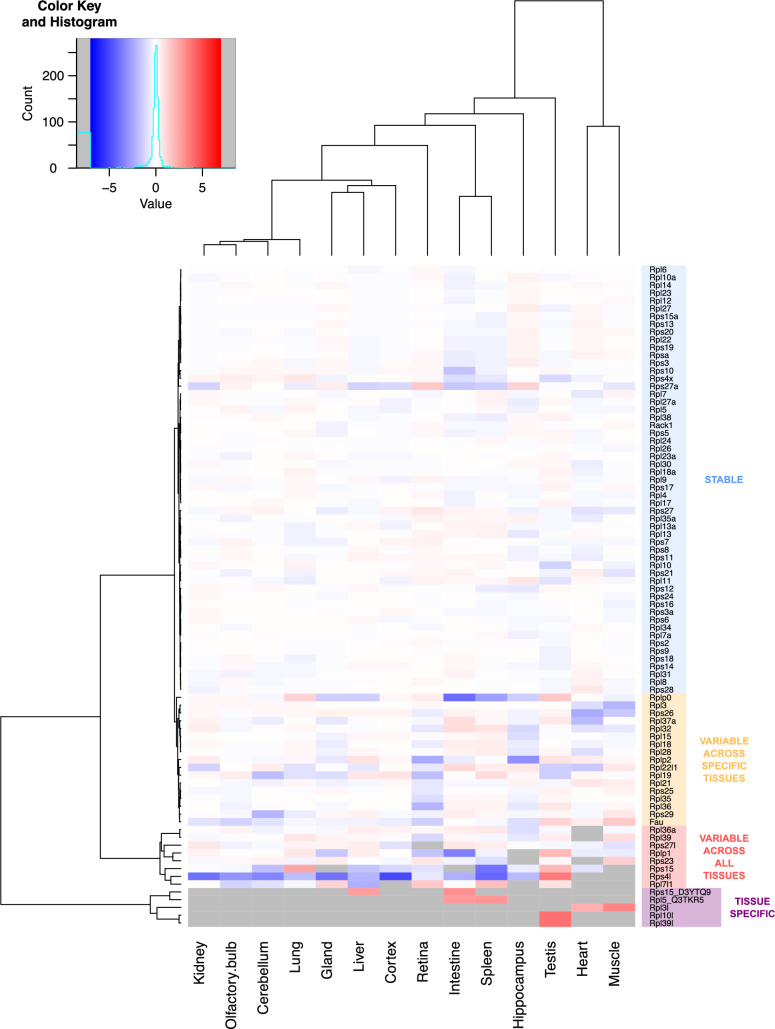
Differential RP composition of ribosomal fraction in adult mouse tissues. Heatmap of the log-transformed relative abundance normalized to the sum of all RPs for the 85 RPs detected in the ribosomal fraction of all tissues, from depleted (in blue) to enriched (in red). Grey boxes represent no detection of the RP in the ribosomal fraction of the corresponding tissue

Heterogeneity in RPs distribution among the ribosomal fractions of different tissues was visualized by representing the relative abundance of each RP in the different tissues normalized by the sum of abundances of all RPs (Fig. 3). Hierarchical clustering revealed that the majority of RPs are invariable among tissues, most of them being canonical RPs belonging to both the large and small ribosomal subunits (e.g. Rps2, Rps6, Rpl22, Rpl10a). In contrast, we also highlighted variable RPs across tissues. These variable RPs can be divided into three groups: one group containing most paralogs of canonical RPs (e.g. Rpl3l, Rpl10l, Rpl39l) that were specifically detected in one or two tissues, one group with high variability across most tissues (e.g. Rps15, Rplp1, Rpl39), and one group with variability in specific tissues (e.g. Rps26, Rps29, Rplp2) (Fig. 3).

**Fig. 4:**
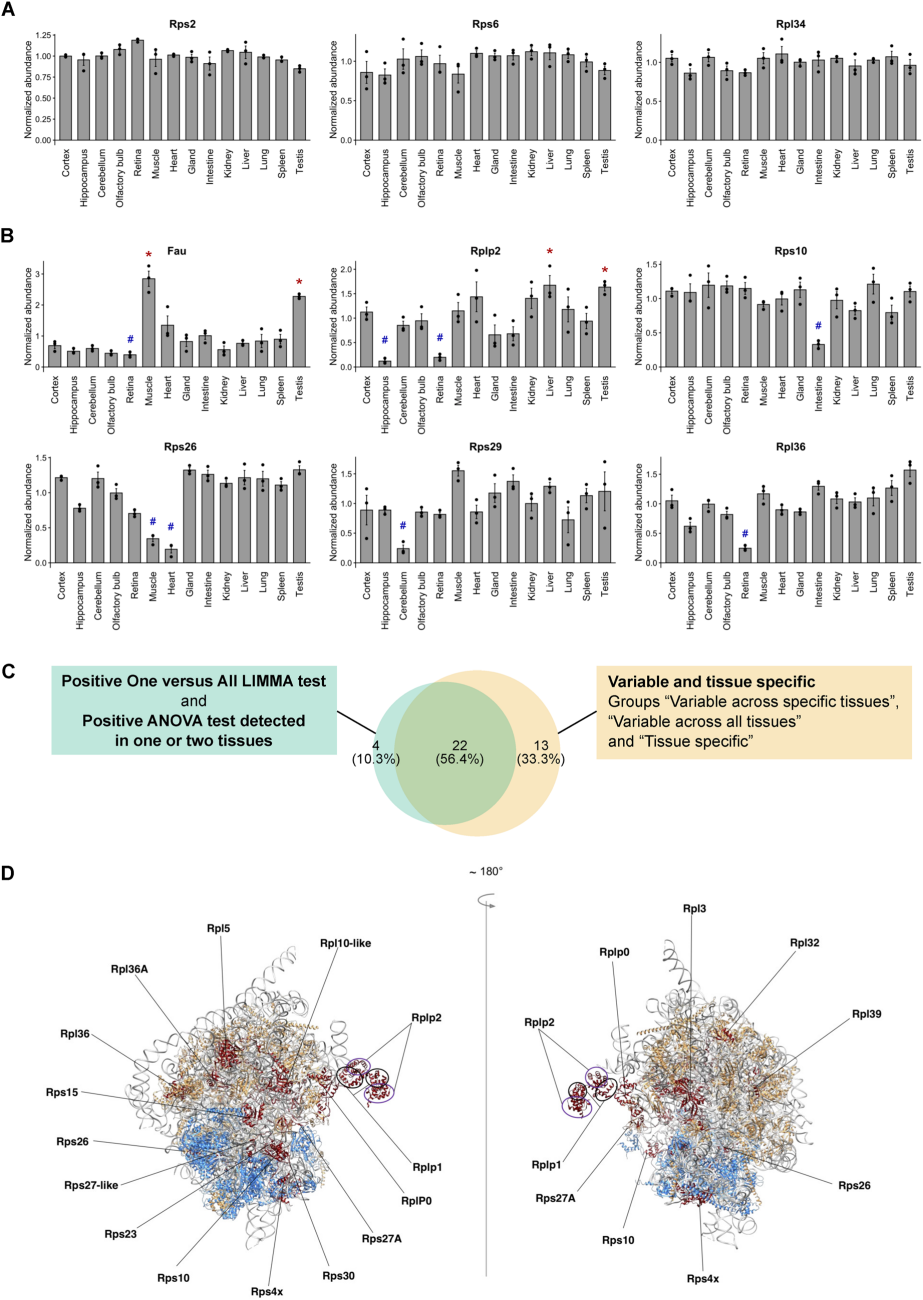
MS-based label-free quantitative proteomics shows differential abundance of individual RPs in ribosomal fractions of adult mouse tissues. **A** Barplot representation of the relative abundance in each tissue of stable RPs normalized to the sum of all RPs (Rps2, Rps6 and Rpl34 serve as examples). The mean ± s.e.m. is plotted for each tissue, as well as values of individual replicates. **B** Barplot representation of the relative abundance in each tissue of variable RPs normalized to the sum of all RPs (Fau, Rplp2, Rps10, Rps26, Rps29 and Rpl36 serve as examples). The mean ± s.e.m. is plotted for each organ, as well as values of individual replicates. *q-value < 0.01 (LIMMA test) and log_2_FC > 1 for One versus All comparisons. ^#^q-value < 0.01 (LIMMA test) and log_2_FC < – 1 for One versus All comparisons. **C** Venn diagram showing overlap of RPs found variable in relative abundance between tissues using statistical tests (green) and hierarchical clustering (yellow). **D** Visualization of molecular structure of the ribosome; positions of variable RPs are indicated

This heterogeneity in RP composition between ribosomal fractions of different tissues was confirmed after statistical analysis of the data. For this purpose, we used an ANOVA test, followed by One versus All LIMMA test (cf. Materials and Methods) for RPs exhibiting ANOVA test q-value < 0.01, allowing us to highlight RPs exhibiting a significant enrichment or depletion in the ribosomal fraction of specific tissues. This strategy revealed 59 proteins with similar abundance in the ribosomal fractions from the different tissues analyzed (ANOVA test q-value > 0.01, or ANOVA test q-value < 0.01 but LIMMA test q-value > 0.01 or abs(log_2_FC) < 1.0) (S2–S3 Tables, Fig. 4A). Among them, 46 were also highlighted in the clustering analysis as stable (Fig. 3, group “Stable”). On the other hand, 26 RPs displayed significant variability across the different tested tissues (ANOVA test q-value < 0.01, LIMMA test q-value < 0.01 and abs(log_2_FC) > 1.0) or tissue specificity (ANOVA test q-value < 0.01, specifically detected in one or two tissues) (Fig. 4B, S3 Fig, S3 Table). Among this group, 22 RPs were also highlighted in the clustering analysis as variable (Fig. 4C, S3A Fig). Results from the hierarchical clustering and the statistical test were similar, strengthening the identification of heterogeneity in RP composition of ribosomes in the different organs and tissues. In the group of variable RPs, we found RPs whose normalized abundances are highly variable across all tissues, e.g. Rps30 (Fau) enriched in muscle and testis and depleted in retina and Rplp2 enriched in liver and testis and depleted in hippocampus and retina (Fig. 4B). We also found RPs whose normalized abundances are significantly different in a subset of specific tissues, e.g. Rps10 less abundant in intestine, Rps26 less abundant in muscle and heart, Rps29 less abundant in cerebellum and Rpl36 less abundant in retina (Fig. 4B, S3 Table).

Depending on the availability of specific antibodies, we verified some of these results by western blot. We were notably able to validate the enrichment of Rps30 (Fau) in the ribosomal fraction of the testis compared to that of the cortex and the retina. Rps26 was found to be similarly abundant in ribosomal fractions prepared from cortex, retina, hippocampus and testis (S3B-C Fig). These results were consistent with those obtained using our discovery proteomic strategy, supporting our findings that some RPs show variable incorporation in the ribosomal fractions of different organs/tissues, while others are stable.

Several paralog RPs have been shown to display tissue-specific transcript expression [50], as well as to control cell type-specific function, e.g. Rpl10l in the testis or Rpl3l in the muscle [33]. Our MS-based quantitative proteomic analysis demonstrated the tissue-specific protein expression and incorporation into ribosomes of these paralogs. Indeed, Rpl3l was uniquely detected in the ribosomal fractions of heart and muscle, while Rpl10l was exclusively detected in the testis ribosomes (S4A Fig). Interestingly, we found that the corresponding canonical RP showed decreased abundances specifically in the tissue in which the paralog version was detected. These results strongly suggest that the paralog replaces the canonical RP within the cytoplasmic ribosome. For example, Rpl3 is two-fold and four-fold less abundant in heart and muscle ribosomes, respectively, compared to all other tissues and Rpl10 is two-fold less abundant in testis ribosomes than in other tissues (S4A Fig).

Finally, as our MS analysis was performed on mixed tissues from males and females, we wonder whether gender may influence RP ribosome composition. To this end, we purified ribosomes from different tissues (liver, cortex and cerebellum) from three males and females and we analysed by western blot the abundance of specific RPs that showed variable levels of incorporation into ribosomes across tissues in our MS-based dataset. For the different RPs tested (Rpl3, Rpl36, Rplp2, Fau, Rsp26 and Rsp6), no significant difference in quantity was observed between males and females in the ribosomal fractions purified from the different tissues (S5A, B Fig). These results suggest that the differential incorporation of specific RPs in different tissues is not sex-dependent.

### Position of variable RPs within the ribosome

Using Chimera software and PDB database (4v6x Human ribosome), we analyzed the localization of RPs showing variable association with the ribosome in the different tissues within the quaternary structure of the complex. Variable RPs appeared to be present either at the periphery of the quaternary structure of the ribosome (solvent side, e.g. Rps15) or at critical functional sites such as the mRNA entry site (e.g. Fau, Rps10) or the tRNA binding sites: aminoacyl (A)-site (e.g. Rpl3/Rpl3l), the peptidyl (P)-site (e.g. Rpl10/Rpl10l), the exit (E)-site (e.g. Rps26), the nascent polypeptide exit tunnel (e.g. Rpl39) and the ribosomal stalk (Rplp0, Rplp1, Rplp2) (Fig. 4D).

Paralog versions of RPs substitute for the corresponding canonical versions in the quaternary structure of ribosomes, where it may sustain a cell type-specific control of translation. In mouse, the paralogous Rpl10l and the canonical Rpl10 differ by three amino acids in their sequences (S4B Fig), producing nearly identical 3D conformations (S4C Fig). Interestingly, we observed that Rpl10l occupies the exact same place as Rpl10 in the quaternary structure of the ribosome (S4D Fig). This suggests that a single ribosome cannot contain Rpl10 and Rpl10l at the same time, pointing towards a specialization of the ribosome through specific RP composition and suggesting downstream control of translation in different cell types and tissues. In drosophila, such paralog-switching events are a hallmark of adult gonads [56], with unique, non-overlapping functions of the paralogous and the canonical versions, as it was shown for Rpl22 and Rpl22-like [57].

### Targeted proteomics-based quantification of RPs in adult tissues

**Fig. 5:**
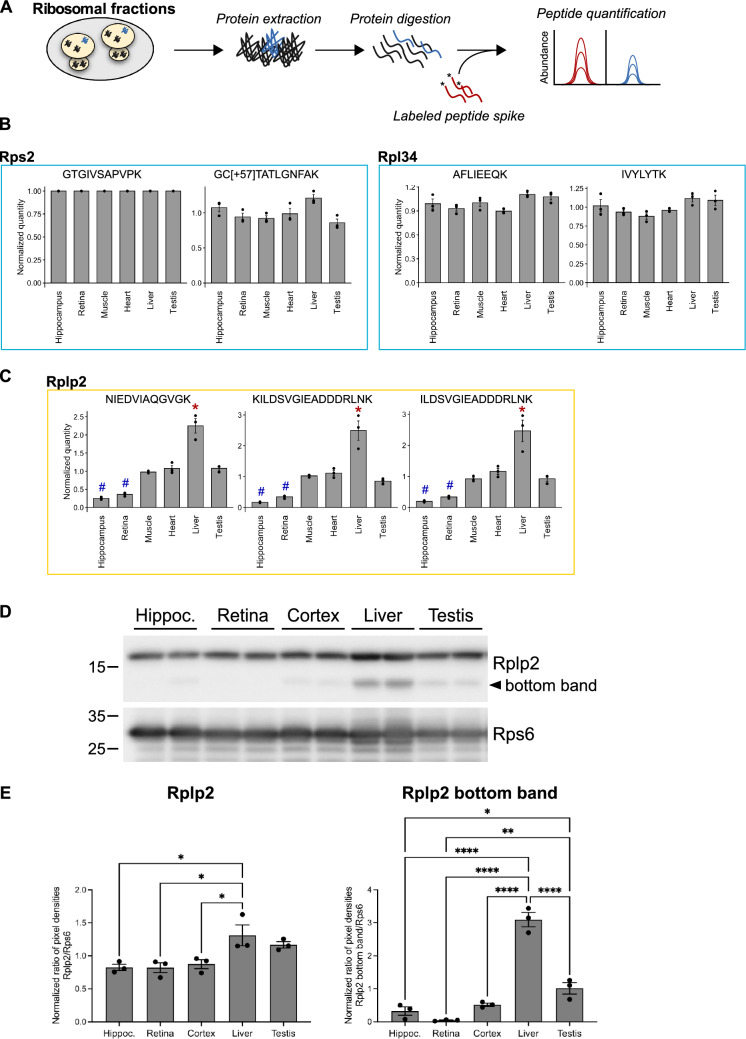
Validation of variable RPs using targeted proteomics. **A** Workflow of targeted proteomic quantification of peptides from specific proteins in ribosomal fractions of selected tissues. **B** Barplot representation of the relative abundance of stable RP-specific peptides (Rps2, taken as a reference, and Rpl34). **C** Barplot representation of the relative abundance of Rplp2-specific peptides. The mean ± s.e.m. is plotted for each organ, as well as values of individual replicates. *q-value < 0.01 (LIMMA test) and log_2_FC > 1 for One versus All comparisons. ^#^q-value < 0.01 (LIMMA test) and log_2_FC < -1 for One versus All comparisons. **D** Western blot analysis of Rplp2 and Rps6 in the ribosomal fractions obtained from the hippocampus, retina, cortex, liver and testis. Each well represents one biological replicate. **E** Barplot representation of the pixel density ratio of Rplp2 and Rplp2 bottom band over Rps6, normalized to the average of all samples. For each tissue/organ, N = 3 biological replicates. One-way ANOVA test with Tukey correction for multiple comparisons, *p-value < 0.05, **p-value < 0.01, ****p-value < 0.0001

To further confirm the specific RP signature of adult organs highlighted in the label-free, unbiased proteomic approach, we performed a targeted profiling of selected RPs to analyze the ribosomal fractions from different tissues. We selected proteins from the stable group (Rps2 and Rpl34), paralogous/canonical RPs with balanced enrichment in specific tissues (Rpl3/Rpl3l, Rpl10/Rpl10l, Rpl39/Rpl39l pairs) and four RPs from the 22 proteins highlighted as variable from both the clustering and the statistical approaches (Rps26, Fau, Rpl36 and Rplp2) (Fig. 4B). We deployed an isotope dilution strategy, using heavy isotope-labelled peptides whose sequences were designed based on the detection of RP-specific peptides in our label-free approach (Fig. 5A). In total, 29 peptides were targeted in the different tissues (S4 Table). To be able to compare the abundances of the different targeted peptides and proteins across the different samples, we used the Rps2-derived peptide GTGIVSAPVPK as normalization reference since Rps2 was found to be stable in the ribosomal fractions across different analyzed tissues (Fig. 4A). Of note, four selected peptides gave noisy data for which no conclusion could be drawn. Additionally, for some RPs, we observed that the absolute quantity in amol inferred from the ratio between heavy and endogenous peptide signals could vary substantially among their different peptides (S4 Table). This is most probably due to uneven solubilization of heavy isotope-labelled peptides, variability in digestion efficiency or limited accuracy of the heavy peptide dosage provided by the manufacturer. Nonetheless, when averaged across all selected organs, we found a high consistency among relative amounts of the measured proteins, as detailed below.

We focused our statistical analysis on a subset of tissues (hippocampus, retina, muscle, heart, liver and testis) for which the set of RPs analyzed by targeted proteomics showed significant variations in the label-free approach. Using the targeted approach, five peptides corresponding to Rpl3l, Rpl10l or Rpl39l were specifically detected in only one or two tissues. The 19 other peptides were quantified in the six tissues. To decipher any significant difference in abundance between the different tissues, we performed an ANOVA test on the measured amounts of these 19 peptides. From this, nine peptides showed no significant change in abundance between different tissues (ANOVA test q-value > 0.01, S5 Table). As expected, peptides belonging to Rps2 and Rpl34 showed no significant change between tissues (ANOVA test q-value > 0.01, Fig. 5B, S5 Table), confirming the stable embedding of Rps2 and Rpl34 in ribosomes from all tissues, as observed in the label-free approach (Fig. 4A). For the 10 peptides showing significant changes between tissues (ANOVA test q-value < 0.01, S5 Table), we further conducted a One versus All LIMMA test to highlight which tissues had a significant enrichment or depletion of each of the corresponding proteins in their ribosomal fraction. For nine of them, a significant depletion or enrichment was shown (comparison tissue versus all others LIMMA test q-value < 0.01 and abs(log_2_FC) > 1.0, S5 Table). They belong to four different proteins: Rpl10, Rpl3, Rpl36 and Rplp2. Interestingly, among them, Rpl3 and Rpl10 have tissue-specific paralogs, respectively Rpl3l detected only in the ribosomal fraction of muscular tissues and Rpl10l detected only in the ribosomal fraction of testis. Importantly, the canonical forms were found significantly depleted in tissues in which the paralogs were specifically detected (S5 Table, S6A, B Fig). Measurements performed on peptides common to the paralogous and canonical forms showed a stable abundance across all organs (ANOVA test q-value > 0.01, S5 Table, S6A, B Fig). These results confirmed the specific RP signature of the ribosomal fractions in the muscular tissues and the testis, in which Rpl3l/Rpl3 and Rpl10l/Rpl10, respectively, balance each other and act as markers of the RP composition in the corresponding tissues. We confirmed these results for the Rpl3l/Rpl3 pair using western blot analysis on ribosomal fractions purified from liver, cortex and muscle. We found that Rpl3l was detected almost exclusively in ribosomal fractions from muscle, while Rpl3 was significantly less abundant in the ribosomal fraction prepared from this tissue compared to those from liver and cortex (S6C, D Fig).

Finally, our targeted analysis confirmed the variations in abundance of Rplp2 previously observed in ribosomal fractions from different tissues. Indeed, using three different peptides, a significant depletion of Rplp2 was detected in the ribosomal fractions prepared from the hippocampus and the retina, compared to the other organs (ANOVA test q-value < 0.01, comparison one tissue versus all others LIMMA test q-value < 0.01 and log_2_FC < -1.0) and a significant enrichment in the ribosomal fraction of the liver (ANOVA test q-value < 0.01, comparison one tissue versus all others LIMMA test q-value < 0.01 and log_2_FC > 1.0) (Fig. 5C). These results were consistent with those of the label-free approach and further confirmed the differential RP composition of ribosomal fractions in the different tissues. We further analyzed Rplp2 amounts in ribosomal fractions from different tissues using western blot analysis. Two different bands were detected (Fig. 5D) and analyzed both bands detected by an anti-Rplp2 antibody in the ribosomal fractions of cortex, retina, hippocampus, liver and testis. We found a significant enrichment of Rplp2 in the liver compared to brain tissues (cortex, retina and hippocampus) (Fig. 5D, E). Very interestingly, when we focused on the bottom band only (~ 13 kDa), we found that Rplp2 is depleted in the retina, enriched in the liver and intermediate in the cortex and testis (Fig. 5D, E), consistent with our targeted profiling (Fig. 5C). Altogether, this provides a validation of our targeted profiling approach in purified ribosomal fractions of different organs/tissues.

### Correlation of relative transcript expressions and RP composition of ribosomes in adult tissues

To unravel the origin of observed variations in RP content of ribosomal fractions from different adult mouse tissues, we sought to analyze RP expression at the mRNA level in each organ. For this, we used published datasets mouse transcriptome atlas: the transcriptomic BodyMap [58] and the Mouse ENCODE Consortium project [59]. We also use a Human transcriptome atlas (the Illumina Human Body Map (GSE30611)) in order to decipher to which extend our results in mice can be extrapolated to humans. We compared the level of RP incorporation into ribosomes from our proteomic analysis to each of these three mRNA expression dataset as analyzed by [50]. To estimate RP transcript abundances, we computed the reads per kilobase per million mapped reads (RPKM) normalized by the sum of RPKM of all the RPs in each tissue [50]. The relative expression of RPs among organs represented as a heatmap shows little variation at the transcript level, except for the three paralogous RPs: Rpl3l, strongly enriched in the heart and muscle, and Rpl10l and Rpl39l, strongly enriched in the testis (S7 Fig).

We then compared the expression of each RP in the three transcriptomic datasets (“Mouse BodyMap”, “Mouse ENCODE” and “Human Body Map”) with the results of our MS-based quantitative proteomic analysis of ribosomal fractions. We focused on organs and tissues found in all datasets: brain, gland, heart, kidney, liver, lung and testis. For the Mouse ENCODE dataset, no direct data from cortex are available as they generated a brain sample. We decided then to compare values of the brain from that of the cortex.

**Fig. 6:**
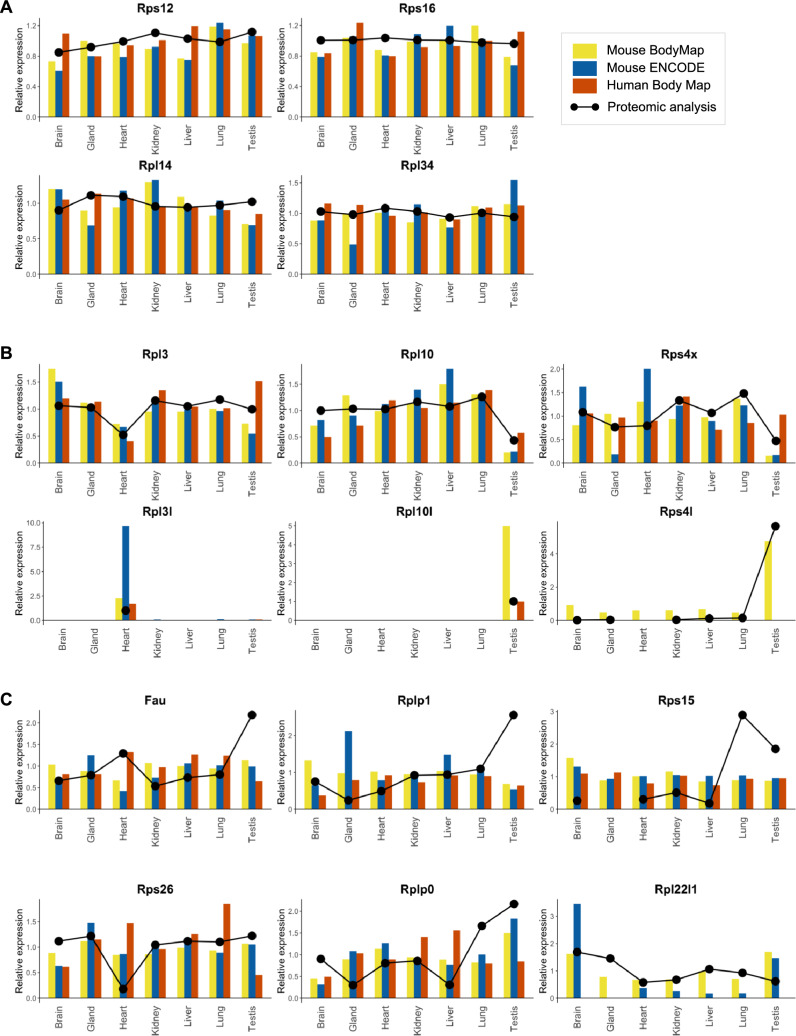
Comparison of the relative expression of RPs in transcriptomic-based datasets and MS-based proteomic analysis of the ribosomal fraction in different mouse tissues. **A**–**C** Barplot representation of the relative expression of each RP at the transcript level, with data from Mouse BodyMap [58], Mouse ENCODE Consortium [59] and Illumina Human Body Map 2.0 as analyzed by [50]. The relative abundance as determined in our present study is superimposed with black dots and line. **A** Examples of stable RPs with similar transcriptomic and ribosome proteomic profiles (Rps12, Rps16, Rpl14, Rpl34). **B** Examples of paralogous RPs and corresponding canonical forms, with similar transcriptomic and ribosome proteomic profiles (Rpl3l/Rpl3, Rpl10l/Rpl10, and Rps4l/Rps4x). **C** Examples of variable RPs with tissue-specific enrichment or depletion in the ribosomal fraction not reflected by the transcriptomic profile (Fau, Rplp1, Rps15, Rps26, Rplp0, Rpl22l1)

For many of the stable RPs found in our proteomic analysis (59 RPs with non-significant difference in abundance in ribosomal fractions of the different tissues), the corresponding transcript expression did not vary across organs in all transcriptomic datasets, e.g. Rps12, Rps16, Rpl14 and Rpl34 (Fig. 6A), showing an overall good correlation between transcriptomic expression and protein incorporation into ribosomes for this group of RPs. Moreover, several variable RPs detected by our proteomic approach (26 RPs with significant variation in abundance in ribosomal fractions of the different tissues) also showed variability at the transcript level. This is the case of the paralogous RPs Rpl3l, Rpl10l (not reported in the Mouse ENCODE dataset), Rps4l (not reported in the Mouse ENCODE and the Human Body Map datasets) and the corresponding canonical Rpl3, Rpl10 and Rps4x (Fig. 6B). Interestingly, the relative transcript expression of the canonical RPs corresponding to Rpl3l and Rpl10l correlates with the relative abundance measured in purified ribosomes in our proteomic data. Indeed, Rpl3 transcript is less abundant in the heart than in the other organs, while Rpl10 is less abundant in the testis (Fig. 6B).

In contrast, for several variable RPs, we observed little correlation between the relative expression level of transcripts in tissues and the relative abundance of corresponding proteins in the ribosomal fractions. This is the case for Fau and Rplp1, both found enriched in the ribosomal fraction of the testis at the protein level, but measured at similar amounts in the different tissues at the transcript level (Fig. 6C). Similarly, Rps15 was found significantly enriched in ribosomal fractions of the lung and testis compared to other tissues, but its transcript level was found stable across tissues. Another example is Rps26 found in lower protein amounts in the ribosomal fraction of the heart compared to other tested organs/tissues, but found in similar amounts at the transcript level (Fig. 6C). In other cases, transcript levels showed consistent enrichment or depletion in a particular tissue, but with a decorrelation from the abundances measured in ribosomal fractions for the corresponding proteins. This is the case of Rplp0, found enriched in the ribosomal fractions of the lung and the testis, but whose transcript level is consistently depleted in the brain (Fig. 6C). Another example is Rpl22l1, depleted in heart and testis ribosomes, but enriched in the brain and testis at mRNA level.

Altogether, these results indicate that the relative level of transcripts of a given RP does not necessarily correlate with the relative abundance of its corresponding protein in the ribosomal fraction in adult organs/tissues. Even if these conflicting results may be due to the analysis of different biological samples, they bring out interesting hypotheses: (i) differential post-transcriptional regulation of RP protein expression among organs, and/or (ii) differential incorporation of specific RPs into ribosomes among organs. This may lead to an organ-specific control of translation, which remains to be firmly demonstrated.

## Discussion

The ribosome was commonly regarded as an invariable machinery producing proteins from mRNA templates. It was considered as not being directly involved in the regulation of translation or the selection of mRNAs to be translated. Yet, this dogma has been challenged over the past few years, notably by an accumulation of compelling evidence supporting the variability of ribosome composition across different cell types and in various physiological and pathological conditions [60–62].

**Fig. 7:**
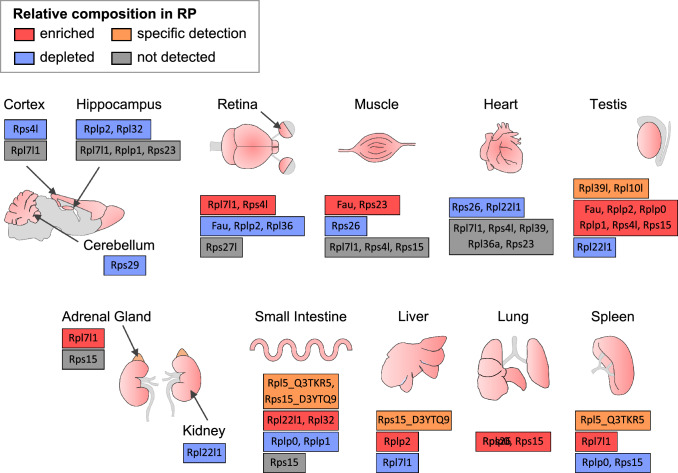
Summary of variations detected for individual RPs in the ribosomal fraction of different mouse tissues and organs. Relative RP enrichment and depletion among the mouse tissues and organs, as well as specific detection and absence of detection, are illustrated for the 22 RPs highlighted as variable from both the clustering and the statistical analyses (see Fig. 4C)

Here, we aimed at deciphering ribosomes’ differential RP composition in 14 different tissues across 11 organs of adult mouse. While the majority of RPs showed no significant difference in abundance in ribosomal fractions prepared from different organs, we found several RPs with variable expression levels. Indeed, some RPs are clearly enriched or, on the other hand, depleted from specific tissues or organs (Fig. 3), which supports the concept of specific ribosomal signatures of adult mouse organs (Fig. 7). This is especially striking given that our macroscopic approach analyzed ribosomal fractions of whole-tissue or whole-organ, strongly suggesting that variations in RP composition likely control organ- or tissue-specific development and function. Similar approach at a whole-organ level in drosophila highlighted specific RP clusters in the 80S fraction of ovary and testis, mainly driven by RP paralog enrichment or switching in the ribosome [56].

We observed that numerous variable RPs identified by our work are located in the peripheral regions of the ribosome. So, we cannot rule out the possibility that these RPs may be detached from the ribosome during sample processing, depending on the tissue. Yet, as we treated all tissues and organs in the same way, we should expect similar behaviors, unless the strength of the interaction of some RPs with the ribosome is different from tissue to tissue. Therefore, we account for their differential detection not by a possible detachment of these RPs during sample preparation, but rather by their actual differential integration into the ribosomal complex. With this assumption, we detected significant differences in ribosome composition among the different organs, suggesting an organ-specific RP signature of the ribosome. As protein translation is a general mechanism of gene regulation, we originally planned the analysis without any consideration toward gender bias, thus samples were generated from mix male/female tissues. To date, no study addressed the possible modulation of ribosome composition depending on sex at a global scale. However, a number of clues suggest possible differences. For example, RPS4 is encoded by sexual chromosomes and its sequences slightly differs between the Y and the X isoforms. Gonad-specific ribosomes have specific compositions and show specialization [52, 56]. Other studies suggested RP differences in expression between males and females, for example upon a stroke [63], however only studying the global RP expression without any information on the composition of the ribosomes. Here, by comparing the relative amounts of specific RPs between males and females using western blot experiments, we found no gender bias for the RPs found to be differentially incorporated into ribosomes of various tissues (S5 Fig). However, we have only analyzed a limited number of RPs. A comprehensive comparison of ribosome composition in tissues from male and female would be highly informative.

With the ambition of determining the absolute amounts of specific RPs within ribosomes, we used a targeted proteomics approach relying on the use of isotopically-labelled peptides. Unexpectedly, we found a major limitation: the peptides quantities determined could differ from a factor of 10 between different measured peptides of the same RP. Even if this issue prevented us from concluding on the precise stoichiometry of each RP within the ribosomal fraction of each tissue, we found great consistency between relative amounts of the different peptides of each RP in the different tissues. These results further confirm the significant differences in RP composition highlighted by the label-free approach, and were validated by western blot analysis for some RPs, when antibodies were available (Fig. 5 and S6A, B Fig). It is interesting to note that other studies using a similar targeted strategy have based their conclusions on the analysis of at least two peptides per protein or of a single peptide with an additional control for specificity of detection [35].

Tissue-specific RP expression may regulate tissue development and homeostasis through regulation of mRNA translation at one or more stages (mRNA selectivity, translation initiation, elongation or fidelity), for example Rpl38 enrichment driving translation of specific HOX-coding mRNAs in axial patterning by facilitating 80S complex formation on these mRNAs [37, 38]. Our proteomics data on various adult mouse tissues support the concept of heterogeneity in ribosome composition, which may underlie the specific regulation of translation. Considering the variability in the RP composition of the ribosomal fraction in the different tissues analyzed, our observations are suggestive of a specialized function of variable (tissue-enriched or tissue-depleted) RPs in the regulation of the translational process to serve specific functions. This feature may be linked to specialized translation that ensures cell type-specific functions, which remains to be demonstrated for many RPs. This is particularly the case of Rpl39l, a paralog of Rpl39, consistently found to be enriched in the testis. A recent study used a proteomics-based approach, similar to ours, to highlight inter-organ variations in 80S monosome composition and showed Rpl39l enrichment in sample prepared from the testis [52]. Even though this analysis yields to different clustering of variable versus stable RPs compared to our results, potentially due to the analysis of different fractions (i.e*.* 80S monosomes and total ribosomes), this study emphasizes that the differential integration of Rpl39l in ribosomes, not only in adult organs, but also over the course of development, has a direct impact on male germ cell function [52]. In another study, Genuth and colleagues addressed the heterogeneity of ribosome composition in human embryonic stem cells following successive stages of cell fate specification [64]. They did so by collecting ribosomes from the polysomal (actively translating ribosomes) fractions [64]. They show that the expression of 31 RPs is modified over the course of cellular differentiation in polysomes, including Rpl10a. They demonstrate that this specific RP controls the expression of genes involved in Wnt signaling and play a key role in paraxial mesoderm lineage. In addition, their study highlighted Rplp2 as a RP whose abundance in the ribosomal fraction progressively diminishes during mesoderm differentiation. Finally, one should also consider that tissue-specific RP enrichment or depletion may impact tissue specification and function through extra-ribosomal functions [75–77]. Altogether, the exact role played by RP modulations of ribosome composition in each organ remains to be clarified, as well as the extent to which heterogeneous populations of ribosomes co-exist in each organ. How these different populations contribute to translational regulation (i.e. which mRNAs are translated by which ribosome populations) and how this leads to organ specification and function will be the next questions to address. More generally, solving the biochemical and gene expression consequences of ribosome composition changes in various contexts is seen as a major challenge [78].

At the cellular level, ribosome heterogeneity may allow cells to respond rapidly to external stimuli by regulating gene expression at the translational level [22]. Whether the intracellular population of ribosome is itself heterogeneous is an important yet technically challenging question to address. It has been tackled by Barna and colleagues in mouse embryonic stem cells [35]. By using MS-based quantitative proteomic approaches to characterize translationally active ribosomes, they found RPs with variable abundances between ribosomes from polysomes and free subunits (e.g. Rpl10a/uL1, Rpl38/eL38, Rps25/eS25), as opposed to invariant RPs. These results support the notion of an heterogeneous population of ribosomes within the cell to sustain various cellular functions via mRNA-specific translation [35]. Here, our study aimed at providing a comprehensive map of ribosome heterogeneity at a global scale between different organs/tissues. For this reason, we chose to consider the totality of the purified ribosomal fraction and exclude any bias towards a particular fraction (i.e. without separating subunits, monosomes and polysomes), which can be considered as a limitation of our study. Investigating the differences in RP composition between such different fractions is of great interest and will undoubtedly be the purpose of future work.

Furthermore, advanced cryo-electron tomography imaging techniques will enable to resolve ribosome heterogeneity at a subcellular level, thus identifying intra-cellular populations of specialized ribosomes, for instance in association with different organelles [65].

Then the next key question is: what mechanisms are at play to control inter-organ variation in RP ribosomal composition? In the present study, we compared our proteomic data to transcriptomic data from available mouse and human wide RNA-sequencing datasets. The transcript and protein relative expressions correlate well for many RPs among the different organs. This is particularly the case of paralogous RPs Rpl3l, Rpl10l and Rps4l, and the respective canonical RPs Rpl3, Rpl10 and Rps4x that, in addition, show organ-specific compensations at the transcript level as well as at the protein level. Interestingly, this phenomenon of regulation has already been demonstrated for the pair Rpl22/Rpl22l1, where Rpl22 itself has been shown to repress the translation of Rpl22l1 by binding to an hairpin structure of its mRNA [66]. The finding that the paralogous version of a RP competes with the canonical version to incorporate into the ribosome reinforces the hypothesis of specific regulation of the ribosome composition. Several studies describe that such a “paralogous RP signature” ensures specific functions through selective translation, e.g. muscle function [32, 67] or gonad specification and function [56, 68]. In yeast, the balance between two RP forms controls proper mitochondrial function [69] and response to stress [70].

These variations may also have a direct impact on organ-specific functions and their associated defects observed in some ribosomopathies. Indeed, most ribosomopathies are associated to tissue-specific dysfunctions and/or malformations [71, 72]. For example, patients affected by Diamond Blackfan anemia may develop malformations (craniofacial defects) and cardiac defects. Another example is Shwachman-Diamond Syndrome (SDS), a pathology in which patients may exhibit pancreatic dysfunction. In light of our present study, it is possible that, in ribosomopathies, a mutation in an individual RP may underlie organ-specific phenotypes because of its specificity for that particular organ.

An important point is to define how the expression of individual RPs is specifically controlled and what the upstream regulatory mechanisms are. Regulation of expression of individual RPs may occur at the transcription level through the control of cell type-specific transcription factors. Indeed, studying RP gene expression in different human tissues, Guimaraes and colleagues identified transcription regulatory elements located in the promoter of RP genes, suggesting that lineage-specific regulations may be at work through the activity of particular transcription factors [51]. Although little difference is observed in promoter utilization in cell type-specific versus non-specific RPs and despite the heterogeneity in promoter regulatory sequences of individual RPs, it is possible that a set of cell type-specific transcription factors orchestrates RP gene expression [51, 73]. This accounts for the level of heterogeneity observed at the transcript level, where mechanisms of co-regulation of RPs expression remain to be determined [37].

In a meta-analysis of several transcriptomics-based and translatomics-based studies on humans and rodents, Panda et al. highlighted organ-, stage- and tumorigenic state-specific RP signatures [74]. Interestingly, they found a high correlation between ribosome-protected footprints and mRNA levels for mRNA coding for RPs, notably in liver, brain, hippocampus and heart [74], suggesting correspondence between RP mRNA and protein levels. However, taking into account extra-ribosomal functions of RPs [75] and actual integration of proteins in the functional ribosomal complex is crucial to further illustrate ribosome heterogeneity in terms of RP composition. Our study reveals that several RPs display no or little correlation between the relative transcript expression level and the relative protein abundance among different mouse tissues (Fig. 6C). Similarly, in Drosophila, the inter-organ differences in RP composition are not simply driven by variations in RP mRNA levels [56]. This observation brings out several hypotheses, such as a differential post-transcriptional regulation of RP protein expression among tissues and/or a differential incorporation of RPs into the ribosome.

Our results also pave the way for the key questions, as yet to solve, of how and where the cell fine-tunes the ribosome composition to regulate translation. Of note, in our study, Rplp0, Rplp1 and Rplp2 (RPs of the ribosomal stalk, a lateral protuberance of the ribosome that binds translation factors) were found variable in both the clustering and the statistical approaches. These RPs also show inter-tissue variability at the mRNA level [79]. Interestingly, free Rplp1 and Rplp2 integrate into the ribosome in the cytoplasm, and this association regulates the translational activity of the ribosome [80]. More recent studies have demonstrated the local protein synthesis and integration of individual RPs in the ribosome of distal neuronal compartments [81, 82], pointing towards a local remodeling of ribosome RP composition during neuronal circuit development or in response to stress.

To conclude, our study adds up to the growing evidence that diverse cell types exhibit specific – and probably specialized – RP expression in a mammalian organism. Based on MS-based quantitative proteomic data, our work brings firm evidence that this tissue-specificity occurs not only at the protein expression level, but also at a ribosome integration level. Comparison with transcriptomic datasets shows that these specific RP signatures do not necessarily correlate with RP mRNA levels, opening questions about inter-tissue ribosome heterogeneity regulatory mechanisms.

## Material and methods

### Tissue sampling and ribosome purification by subcellular fractionation

Wild-type (WT) C57BL/6J adult mice were used in this study, regardless of their sex, except for collection of the testis. Organs and tissues of 4–6 week-old mice were dissected and flash-frozen in liquid nitrogen. Ribosomal fraction purification was performed according to [54]. All steps were performed on ice or at 4 °C. Samples were lysed in freshly prepared buffer A (50 mM Tris HCl pH 7.4, 250 mM sucrose, 250 mM KCl, 5 mM MgCl_2_ (Sigma-Aldrich)) using a Cell Mill (RETSCH MM 400). An aliquot of the cell suspension (total fraction) was saved for SDS-PAGE. IGEPAL detergent (Sigma-Aldrich) was added to the remaining volume to a final concentration of 1%. After 20 min incubation on ice, the lysate was centrifuged at 750 × *g* to pellet nuclei (nuclear fraction) then 12 500 × *g* to pellet mitochondria (mitochondrial fraction). The supernatant (post-mitochondrial fraction) was loaded on a sucrose cushion (1.25 M sucrose, 0.25 M KCl, 5 mM MgCl_2_, 50 mM Tris–HCl pH 7.4) and ultracentrifuged at 250 000 × *g* for 2 h (Beckman Optima TL 100 Ultracentrifuge). After ultracentrifugation, 50 µl of supernatant (cytoplasmic fraction) was saved. The ribosome pellet was washed twice in ultrapure ice-cold water and resuspended either in 50 µl of Laemmli buffer (ribosomal fraction) or in buffer C (tris HCl 50 mM pH 7.4; 5 mM MgCl_2_, 25 mM KCl). 0.5 μl benzonase (Sigma-Aldrich) was added to the nuclear sample and incubated for 10 min at 37 °C to digest DNA.

### Protein quantification

After denaturation at 95 °C for 5 min, the protein concentration of all fractions was determined using Pierce BCA Protein Assay Kit (ThermoFisher Scientific).

### Western blot

20 µg of protein were loaded on a 12% SDS-PAGE gel and separated by electrophoresis for 4 h at 230 V. Proteins were then transferred on nitrocellulose membrane (Thermo Scientific) under a constant amperage of 250 mA for 3 h. Membrane was blocked in fat free 5% milk powder in Tris-Buffered Saline (TBS) and incubated with the following primary antibodies diluted in 5% fat free milk powder in TBS with 0.1% tween (TBS-T) overnight under agitation at 4 °C: anti-Rpl22 (1:2000, mouse, Santa Cruz sc-373993), anti-Rps6 (1:5000, rabbit, Cell Signaling 2217), anti-Fau (1:500, Proteintech 13,581-1-AP), anti-Rplp2 (1:1000, Invitrogen PA527541), anti-Rps26 (1:500, Proteintech 14,909-1-AP), anti-Rpl3 (1:2000, Proteintech 11,005-1-AP), anti-Rpl3l (1:500, Antibodies Online ABIN2706985), anti-Rpl36 (1:500, Proteintech 15,145-1-AP), anti-H3 (1:10 000, Cell Signaling 9715), anti-HSP60 (1:2000, Santa Cruz sc-376240), anti-GAPDH (1:5000, Proteintech 60,004-1-Ig). On the following day, membranes were washed and then incubated with horseradish peroxidase-linked secondary antibodies: anti-rabbit (Proteintech SA00001-2) or anti-mouse (Millipore 12–349) diluted to 1:5000 or 1:10 000 in TBS-T. Membranes were probed with ECL substrate (100 mM Tris HCl pH 8.5, 0.5% coumaric acid (Sigma-Aldrich), 0.5% luminol (Sigma-Aldrich) and 0.15% H_2_O_2_ (Sigma-Aldrich)). Chemiluminescence was visualized with the ChemiDoc system (Bio-Rad).

### Western blot analysis

For quantification of RP abundance on western blots, we quantified the pixel density using Fiji. Each value was normalized to the value of Rps6 abundance blotted on the same membrane. For males versus females comparison, these ratios were normalized to the average RP abundance across female samples of a single organ for each blot. For males versus females comparison, we conducted multiple unpaired t-tests with FDR correction (two-stage step-up method of Benjamini, Krieger and Yekutieli). The FDR-corrected p-value (q-value) is indicated on each plot. For inter-organ comparison, the pixel density ratios (over Rps6) were normalized to the average RP abundance across samples of the entire blot. For inter-organ comparison, we conducted a one-way ANOVA test with Tukey correction for multiple comparisons.

### Coomassie staining

10 µg of protein were loaded on 12% SDS-PAGE gel and separated by electrophoresis for 4 h at 230 V. Gels were fixed in fixing solution [50% methanol (VWR Chemicals), 10% glacial acetic acid (VWR Chemicals)] for 1 h with gentle agitation. After fixation, gels were incubated in staining solution (0.1% Coomassie Brilliant Blue R-250 (Bio-Rad), 50% methanol, 10% glacial acetic acid) for 20 min with gentle agitation. Gels were then washed several times with destaining solution (40% methanol, 10% glacial acetic acid). Gels were imaged when the gel’s background was fully distained.

### Discovery proteomics

#### Mass spectrometry-based analysis

Proteins (between 5 and 10 µg) from tissues preparations were solubilized in Laemmli buffer before loading on top of a 4–12% NuPAGE gel (Life Technologies), stained with R-250 Coomassie blue (Bio-Rad) and in-gel digested using modified trypsin (sequencing grade, Promega) as previously described [83]. The dried extracted peptides were resuspended in 5% acetonitrile and 0.1% trifluoroacetic acid and analyzed by online nanoliquid chromatography coupled to tandem mass spectrometry (LC–MS/MS) (Ultimate 3000 RSLCnano and the Q-Exactive HF, Thermo Fisher Scientific). Peptides were sampled on a 300 μm 5 mm PepMap C18 precolumn (Thermo Fisher Scientific) and separated on a 75 μm 250 mm C18 column (Reprosil-Pur 120 C18-AQ, 1.9 μm, Dr. Maisch HPLC GmbH). The nano-LC method consisted of a 60 min multi-linear gradient at a flow rate of 300 nl/min, ranging from 5 to 33% acetonitrile in 0.1% formic acid. For all tissues, the spray voltage was set at 2 kV and the heated capillary was adjusted to 270 °C. Survey full-scan MS spectra (m/z = 400–1600) were acquired with a resolution of 60 000 after the accumulation of 10^6^ ions (maximum filling time 200 ms). The 20 most intense ions were fragmented by higher-energy collisional dissociation after the accumulation of 10^5^ ions (maximum filling time: 50 ms). MS and MS/MS data were acquired using the software Xcalibur (Thermo Scientific).

#### Data processing

Data were processed automatically using raw2mzDB converter version 0.9.10. Peaklists were obtained using mzDB-access version 0.8.0 (https://github.com/profiproteomics/mzdb/tree/mzdb-processing_0.8.0) by executing the ‘create_mgf’ command (parameters: MS level = 2, no intensity cut-off, precursor_mz = isolation_window_extracted). Peptides and proteins were identified using Mascot (version 2.6) through concomitant searches against a home-made Mus database, classical contaminants database (homemade) and their corresponding reversed databases. The Mus database is composed from Mus musculus Reference Proteome (UP000000589 from UniProt), ribosomal proteins and their isoforms, and home-selected sequences to identify potential variants from 13 mouse canonic ribosomal protein sequences of special interest (variant obtained by blasting their mRNA sequence against the genome or their protein sequence against the translated genome). Finally, this database has been curated to be non-redundant in protein sequences. Trypsin/P was chosen as the enzyme and two missed cleavages were allowed. Precursor and fragment mass error tolerances were set, respectively, to 10 ppm and 25 mmu. Peptide modifications allowed during the search were: carbamidomethylation (fixed), acetyl (protein N-terminal, variable) and oxidation (variable). The Proline software (version 2.1) [84] was used to merge and filter results for each tissue separately: conservation of rank 1 peptide-spectrum match (PSM) with a minimal length of 7 and a minimal score of 25. PSM score filtering is then optimized to reach a False Discovery Rate (FDR) of PSM identification below 1% by employing the target decoy approach. A minimum of one specific peptide per identified protein group was set. Proline was then used to perform MS1-based label free quantification of the peptides and protein groups from the different samples without cross-assignment activated between tissue but activated only between replicates. Protein iBAQ were computed from specific peptides abundances.

Proteins were filtered out if they were not identified in the three replicates of at least one tissue. Protein values were discarded from a tissue if it was detected in one single replicate of a tissue. For each tissue and replicate, total ribosomal protein iBAQ was used to normalize iBAQ of quantified proteins. After log_2_ transformation of normalized iBAQ, ProStaR [85] was used to impute missing values. POV missing values were imputed with slsa method and MEC ones with a low value. For each sample, this low value was set at the minimum value observed for a protein in this sample. Statistical testing was conducted only on ribosomal proteins using ANOVA with a p-value cut-off allowing to reach a FDR inferior to 1% according to the Benjamini–Hochberg procedure. RPs quantified in one or two tissues were considered tissue-specific. For other proteins, in order to discriminate in which tissue RPs positive to ANOVA were differentially abundant, we used Prostar [85] to run LIMMA test with One versus All as contrast. All data were then merged and p-values corrected with Benjamini–Hochberg procedure. Log_2_(Fold Change (FC) One versus All) were calculated without using MEC imputed values. Proteins were considered as significantly enriched (or respectively depleted) in a tissue if their FDR-adjusted p-value was below 0.01 and their log_2_(FC) > 1 (respectively log_2_(FC) < -1). Proteins were considered as not detected in the tissue if their FDR-adjusted p-value was below 0.01 and no log_2_(FC) could be calculated.

#### Data representation and analysis

Ribosomal proteins were filtered out if they were not identified in the three replicates of at least one tissue. Protein values were discarded from a tissue if it was detected in one single replicate. All plots were generated using R software for data representation and analysis [86]. Scatterplots of RP hits were obtained by plotting the log_10_-transformed protein abundances (iBAQ) normalized to the total iBAQ of all RPs per organ, across biological replicates of each organ. To highlight the relationship between the ribosomal fractions of the different organs, the Pearson’s correlation coefficient was used to compare samples from RP iBAQ values. To generate the heatmap of RPs, iBAQ of each RP of one organ was normalized to sum of all RP iBAQ. For each RP, the relative abundance was computed as the log_2_-transformation of the normalized iBAQ averaged on all organs. To highlight the biological differences between the ribosomal fractions of the different organs, hierarchical clustering was performed by computing the euclidian distance of the log-transformed relative abundances of RP between all samples, with the Ward’s clustering method. Barplots of the relative expression of RPs in the ribosomal fraction of each organ were obtained from the iBAQ normalized to the sum of all RPs, and averaged across all organs.

### Quaternary structures

Crystal structures from human ribosomes were downloaded from PDB (4 × 6v, 6oli). All structures were generated using Chimera software [87], version 1.15c. Labels were manually added.

### Targeted proteomics

#### Mass spectrometry-based analysis

Samples were in-gel digested as described for discovery proteomics. Dried extracted peptides were resuspended in 5% acetonitrile and 0.1% trifluoroacetic acid with a mixture of heavy isotope-labeled peptides from Synpeptide (Shanghai, China) spiked-in. Samples were analyzed by online nanoliquid chromatography coupled to tandem mass spectrometry (LC–MS/MS) (Ultimate 3000 RSLCnano and the Q-Exactive HF, Thermo Fisher Scientific). Chromatographic parameters were the same as described for discovery proteomics. The targeted acquisition method combined two scan events corresponding to a full scan event and a time-scheduled PRM event targeting the precursor ions selected for the pairs of heavy and endogenous peptides in ± 2.7 min elution time windows. The full scan MS spectra (m/z = 400–1600) were acquired with a resolution of 30 000 after the accumulation of 10^6^ ions (maximum filling time 200 ms). The PRM event were acquired with a resolution of 30 000 after the accumulation of 10^6^ ions (maximum filling times varying from 55 to 260 ms depending on the number of peptides to target in each run time range).

#### Data processing

Targeted data were processed with Skyline 4.2. Five best product ions (mono-charged y-type ions) per precursor ion were extracted with 5 ppm tolerance. All matching scans were used. Chromatographic peaks were investigated to manually adjust peak integration boundaries transitions. Peptides whose heavy version was observed with too much variation (coefficient of variation superior to 50%) were discarded. For each tissue, only transitions detected with Signal to Noise higher than 10 and in its three replicates were finally used. Signal at the peptide level was obtained by summing the corresponding transition peak areas for heavy and endogenous peptide. The ratios between the endogen and the heavy peptides were used to determine the mol amount of endogenous peptides. To take into account the variability of ribosomal proteins amount in the samples, all data were normalized by the peptide GTGIVSAPVPK from Rps2. We selected tissues for which variations for the followed peptides were expected from the label-free approach (hippocampus, retina, muscle, heart, liver and testis). For peptides detected in the six tissues, statistical testing was conducted on log_2_ transformed data using an ANOVA with a p-value cut-off allowing to reach a FDR inferior to 1% according to the Benjamini–Hochberg procedure. To discriminate in which tissue peptides positive to ANOVA were differentially abundant, we use Prostar to run LIMMA t-test with One versus All as contrast. All results were merged and p-values corrected with Benjamini–Hochberg procedure. Peptides were considered as significantly enriched (respectively depleted) in a tissue if their FDR-adjusted p-value was below 0.01 and log_2_FC > 1 (respectively log_2_FC < -1).

#### Data representation and analysis

All plots were generated using R software for data representation and analysis [86]. For each individual peptide, the relative abundance was obtained from the mol amount (inferred from the ratio between heavy and endogenous peptide signals) averaged on selected organs.

### Analysis of transcriptomic datasets

To compare the relative expression of RP at the transcript level, we used published dataset of mouse and human transcriptomic atlas of adult organs: the Mouse Transcriptomic BodyMap [58], the Mouse ENCODE Consortium project [59] and the Illumina Human Body Map (GSE30611) as analyzed by [50] (Supplementary Table S6 of the publication). For all datasets, read per kilobase per million mapped reads (RPKM) values were retrieved. For the Mouse BodyMap, only samples from males were selected and RPKM values were averaged by organ. We selected all RPs detected in our dataset and all organs in common to the three transcriptomic datasets and our proteomic dataset, with the only approximation of “Cortex” as “Brain” when not available. We adopted the same normalization strategy as [50] and computed the RPKM normalized to the sum of RPKM of all RP of each organ times the number of RP (RPKM * number RPs / sum(RPKM)). To generate the heatmap, we computed the log_2_-transformation of the normalized RPKM averaged on all considered organs. Barplots of the relative expression of RP transcripts of each organ were obtained from the normalized RPKM values averaged on all organs.

## Supplementary Information

Below is the link to the electronic supplementary material.

Supplementary Figure 1: Validation of ribosomal fraction purification from adult mouse tissues. (A) Western blot analysis of markers of the different fractions obtained in the heart, the kidney and the retina: Histone 3 (nuclear), Hsp60 (mitochondrial and cytoplasmic), Gapdh (cytoplasmic), Rps6 and Rpl22 (ribosomal). (B) Protein profiles of the total and ribosomal fractions of each organ/tissue observed by Coomassie blue staining of proteins after SDS-PAGE. Molecular weights (kDa) are indicated on the left based on protein ladder

Supplementary Figure 2: High consistency between biological replicates of the ribosomal fractions prepared from each tissue. Scatterplots of log-transformed protein abundance of the 85 RPs detected across replicates. The Pearson correlation coefficient is indicated on each plo

Supplementary Figure 3: Variations in RP composition among adult mouse organs and tissues. (A) Barplot representation of the relative abundance of variable RPs normalized to the sum of all RPs. The mean +/- s.e.m. is plotted for each organ, as well as values of individual replicates. * q-value < 0.01 (LIMMA test) and log2FC > 1 for One versus All comparisons. # q-value < 0.01 (LIMMA test) and log2FC < -1 for One versus All comparisons. § specific detection, q-value < 0.01 (ANOVA test). nd: not detected, q-value < 0.01 (LIMMA test) and no log2FC calculable for One versus All comparisons. (B) Western blot analysis of the ribosomal fractions obtained from the cortex, the retina, the hippocampus and the testis: Fau, Rps26 and Rps6. Each well represents one biological replicate. (C) Barplot representation of the pixel density ratio of Fau and Rps26 over Rps6, normalized to the average of all samples. For each tissue/organ, N=3 biological replicates. One-way ANOVA test with Tukey correction for multiple comparisons, * p-value<0.05

Supplementary Figure 4: Paralogous RPs and corresponding canonical RPs show balanced enrichment in specific organs. (A) Barplot representation of the relative abundance of Rpl3l, Rpl3, Rpl10l and Rpl10 normalized to the sum of all RPs. The mean +/- s.e.m. is plotted for each organ, as well as values of individual replicates. # q-value < 0.01 (LIMMA test) and log2FC < -1 for One versus All comparisons. § specific detection, q-value < 0.01 (ANOVA test). (B) Alignment of amino acid sequences of mouse Rpl10l and mouse Rpl10. (C) 3D schematic representation of the molecular structure of Rpl10l and Rpl10. (D) Visualization of the localization of Rpl10l and Rpl10 in the molecular structure of the ribosome

Supplementary Figure 5: Specific RPs show no significant difference in incorporation into ribosomes from females and males. (A) Western blot analysis of the ribosomal fractions obtained from the cortex, the cerebellum and the liver, comparing samples from males versus females: Rpl3, Rpl36, Rplp2, Fau, Rps26 and Rps6. Each well represents one biological replicate. (B) Barplot representation of the pixel density ratio of the RP of interest over Rps6, normalized to the average of female samples. For each RP and each tissue/organ, N=3 biological replicates. For each RP, multiple unpaired t-tests with FDR correction (two-stage step-up method of Benjamini, Krieger and Yekutieli) were conducted. FDR-corrected p-values (q-values) are indicated on each plot

Supplementary Figure 6: Targeted proteomics validates differential abundance of corresponding paralogous and canonical RPs in ribosomal fractions of adult mouse tissues. (A) Barplot representation of the relative abundance in selected tissues of peptides shared by or specific to paralogous Rpl10l and to its corresponding canonical form Rpl10. (B) Barplot representation of the relative abundance in selected tissues of peptides shared by specific to paralogous Rpl3l and to its corresponding canonical form Rpl3. The mean +/- s.e.m. is plotted for each organ, as well as values of individual replicates. # q-value < 0.01 (LIMMA test) and log2FC < -1 for One versus All comparisons. § specific detection. (C) Western blot analysis of the ribosomal fractions obtained in the liver, the cortex and the muscle: Rpl3, Rpl3l, Rps6. Each well represents one biological replicate. (D) Barplot representation of the pixel density ratio of Rpl3 and Rpl3l over Rps6, normalized to the average of all samples. For each tissue/organ, N=3 biological replicates. One-way ANOVA test with Tukey correction for multiple comparisons, ** p-value<0.01

Supplementary Figure 7: Relative expression of RP transcripts across adult mouse tissues. Heatmap of the log-transformed relative expression of RPs detected by RNA-sequencing, from depleted in blue to enriched in red. Grey boxes represent no detection of the RP transcript in the corresponding organ. Expression values from the Mouse Transcriptomic Body Map dataset (58)

Supplementary file8 (XLSX 13057 KB)

Supplementary file9 (XLSX 34 KB)

Supplementary file10 (XLSX 39 KB)

Supplementary file11 (XLSX 28 KB)

Supplementary file12 (XLSX 23 KB)

## Data Availability

The discovery LC–MS/MS data have been submitted to the ProteomeXchange Consortium via the PRIDE [88] partner repository under dataset identifier PXD044060. Supp tables can be downloaded by reviewer: https://filesender.renater.fr/?s=download&token=0101839b-60d1-4cba-bc9f-6640e7ba7e89
