# Supplementary figures and images for: Proteomics-based characterization of ribosome heterogeneity in adult mouse organs
Source: Cell Mol Life Sci. 2025 Apr 24;82(1):175. doi: 10.1007/s00018-025-05708-7 (PMC12022211; doi:10.1007/s00018-025-05708-7)

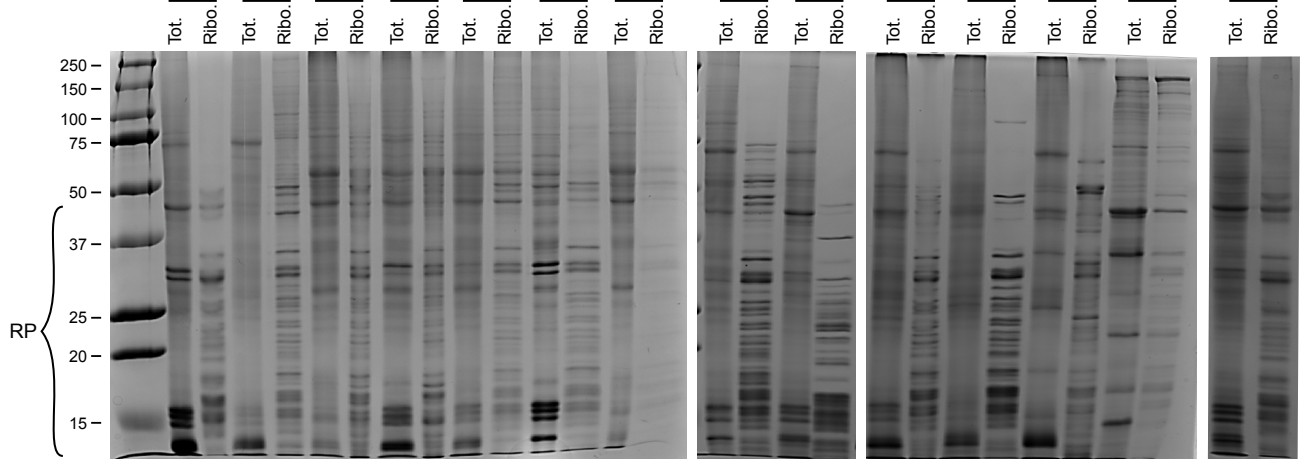

Supplement: Supplementary file 1 — Supplementary Figure 1: Validation of ribosomal fraction purification from adult mouse tissues. (A) Western blot analysis of markers of the different fractions obtained in the heart, the kidney and the retina: Histone 3 (nuclear), Hsp60 (mitochondrial and cytoplasmic), Gapdh (cytoplasmic), Rps6 and Rpl22 (ribosomal). (B) Protein profiles of the total and ribosomal fractions of each organ/tissue observed by Coomassie blue staining of proteins after SDS-PAGE. Molecular weights (kDa) are indicated on the left based on protein ladder [file 18_2025_5708_MOESM1_ESM.pdf]

**Supplementary Figure 2**

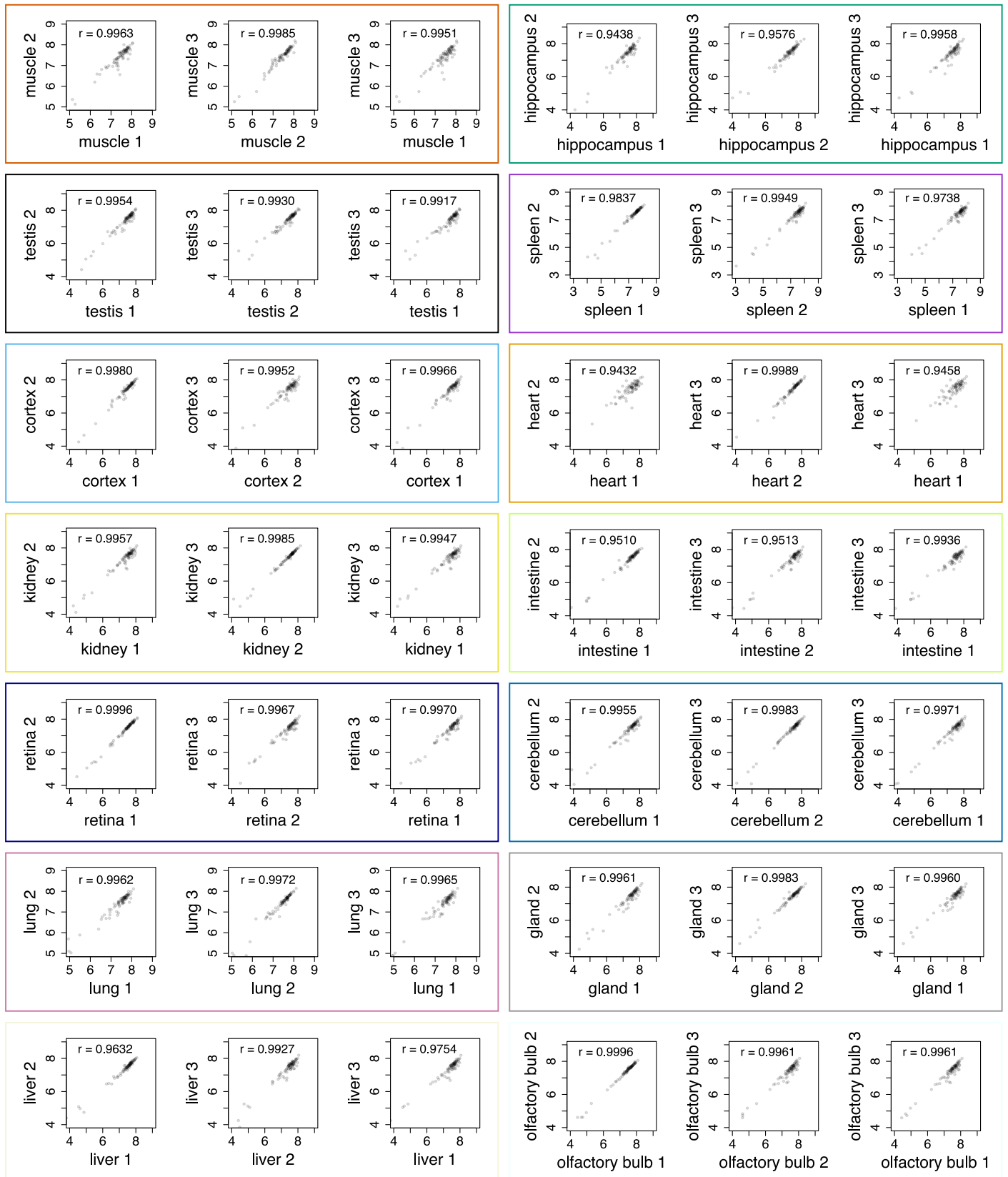

Supplement: Supplementary file 2 — Supplementary Figure 2: High consistency between biological replicates of the ribosomal fractions prepared from each tissue. Scatterplots of log-transformed protein abundance of the 85 RPs detected across replicates. The Pearson correlation coefficient is indicated on each plo [file 18_2025_5708_MOESM2_ESM.pdf]

Supplementary Figure 3

A

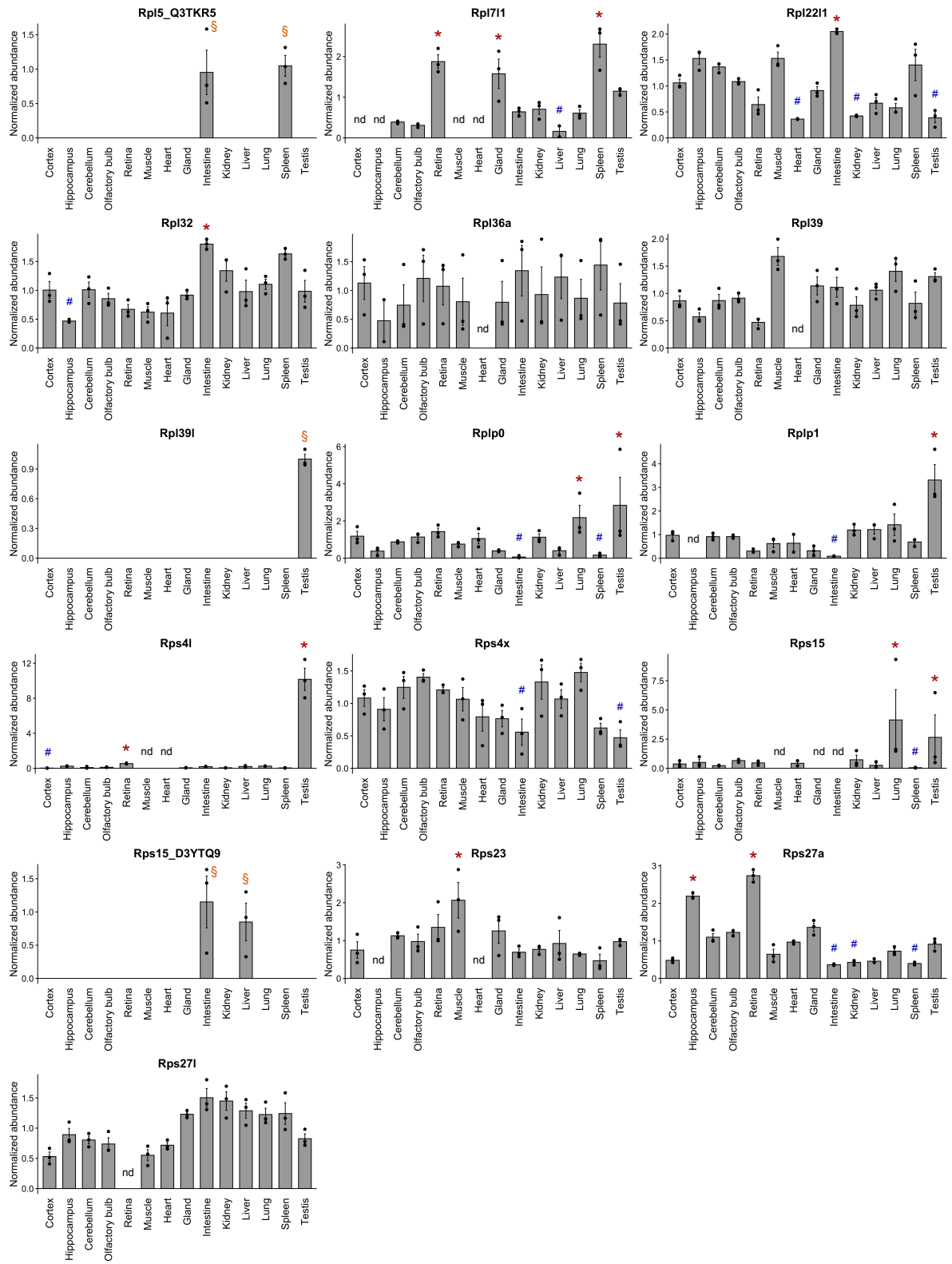

B

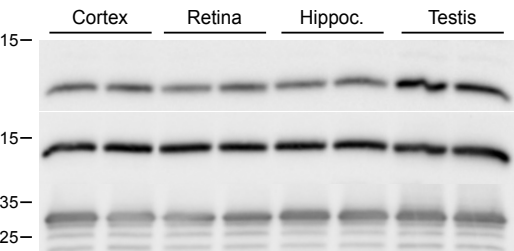

C

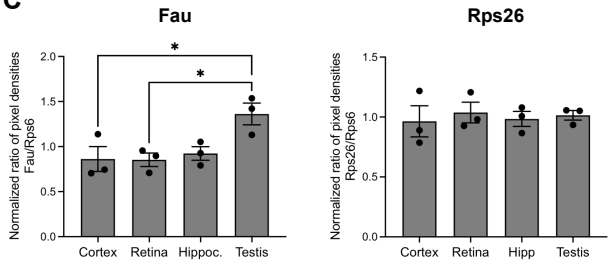

Supplement: Supplementary file 3 — Supplementary Figure 3: Variations in RP composition among adult mouse organs and tissues. (A) Barplot representation of the relative abundance of variable RPs normalized to the sum of all RPs. The mean +/- s.e.m. is plotted for each organ, as well as values of individual replicates. * q-value < 0.01 (LIMMA test) and log2FC > 1 for One versus All comparisons. # q-value < 0.01 (LIMMA test) and log2FC < -1 for One versus All comparisons. § specific detection, q-value < 0.01 (ANOVA test). nd: not detected, q-value < 0.01 (LIMMA test) and no log2FC calculable for One versus All comparisons. (B) Western blot analysis of the ribosomal fractions obtained from the cortex, the retina, the hippocampus and the testis: Fau, Rps26 and Rps6. Each well represents one biological replicate. (C) Barplot representation of the pixel density ratio of Fau and Rps26 over Rps6, normalized to the average of all samples. For each tissue/organ, N=3 biological replicates. One-way ANOVA test with Tukey correction for multiple comparisons, * p-value<0.05 [file 18_2025_5708_MOESM3_ESM.pdf]

Supplementary Figure 4

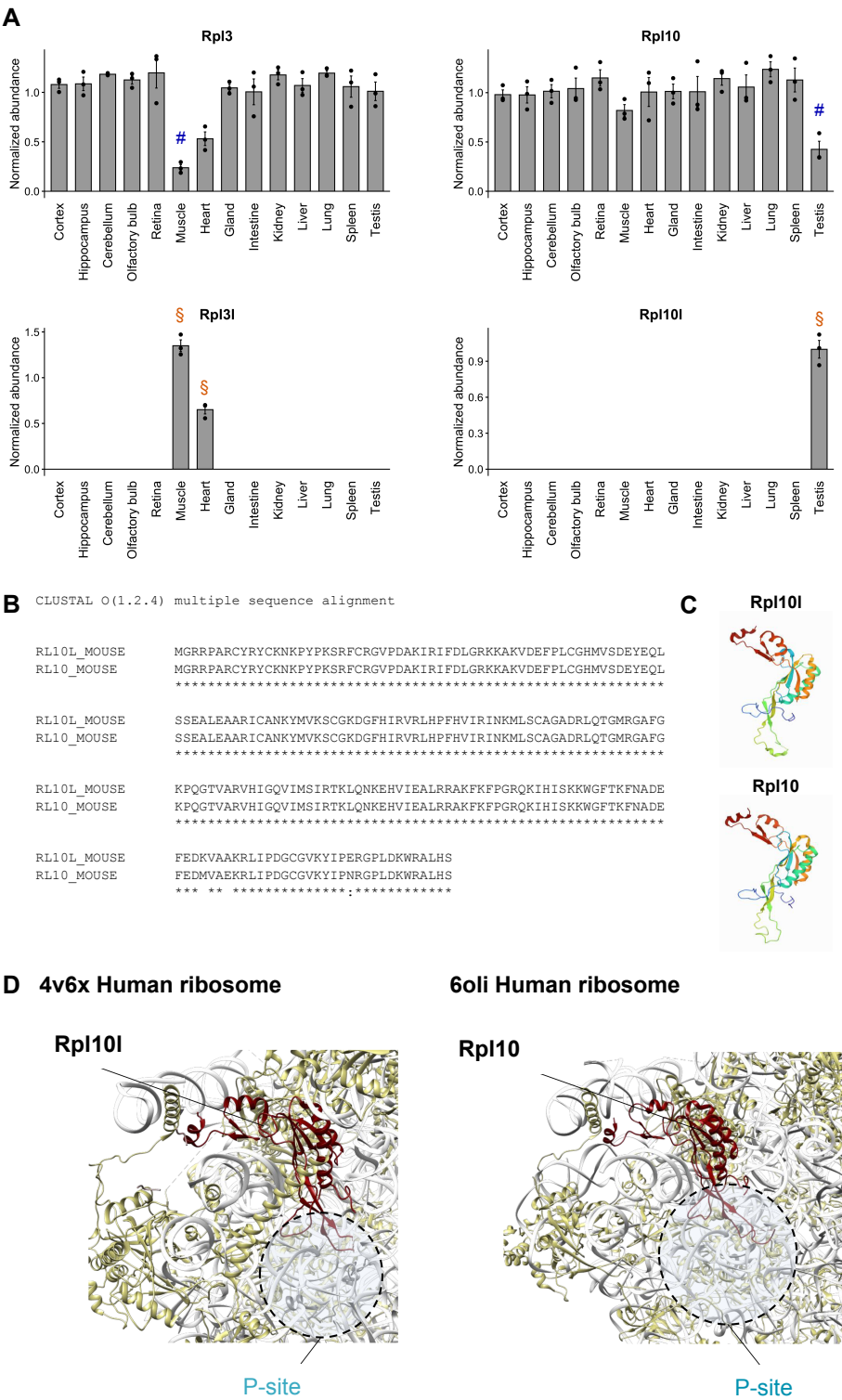

Supplement: Supplementary file 4 — Supplementary Figure 4: Paralogous RPs and corresponding canonical RPs show balanced enrichment in specific organs. (A) Barplot representation of the relative abundance of Rpl3l, Rpl3, Rpl10l and Rpl10 normalized to the sum of all RPs. The mean +/- s.e.m. is plotted for each organ, as well as values of individual replicates. # q-value < 0.01 (LIMMA test) and log2FC < -1 for One versus All comparisons. § specific detection, q-value < 0.01 (ANOVA test). (B) Alignment of amino acid sequences of mouse Rpl10l and mouse Rpl10. (C) 3D schematic representation of the molecular structure of Rpl10l and Rpl10. (D) Visualization of the localization of Rpl10l and Rpl10 in the molecular structure of the ribosome [file 18_2025_5708_MOESM4_ESM.pdf]

**Supplementary Figure 5**

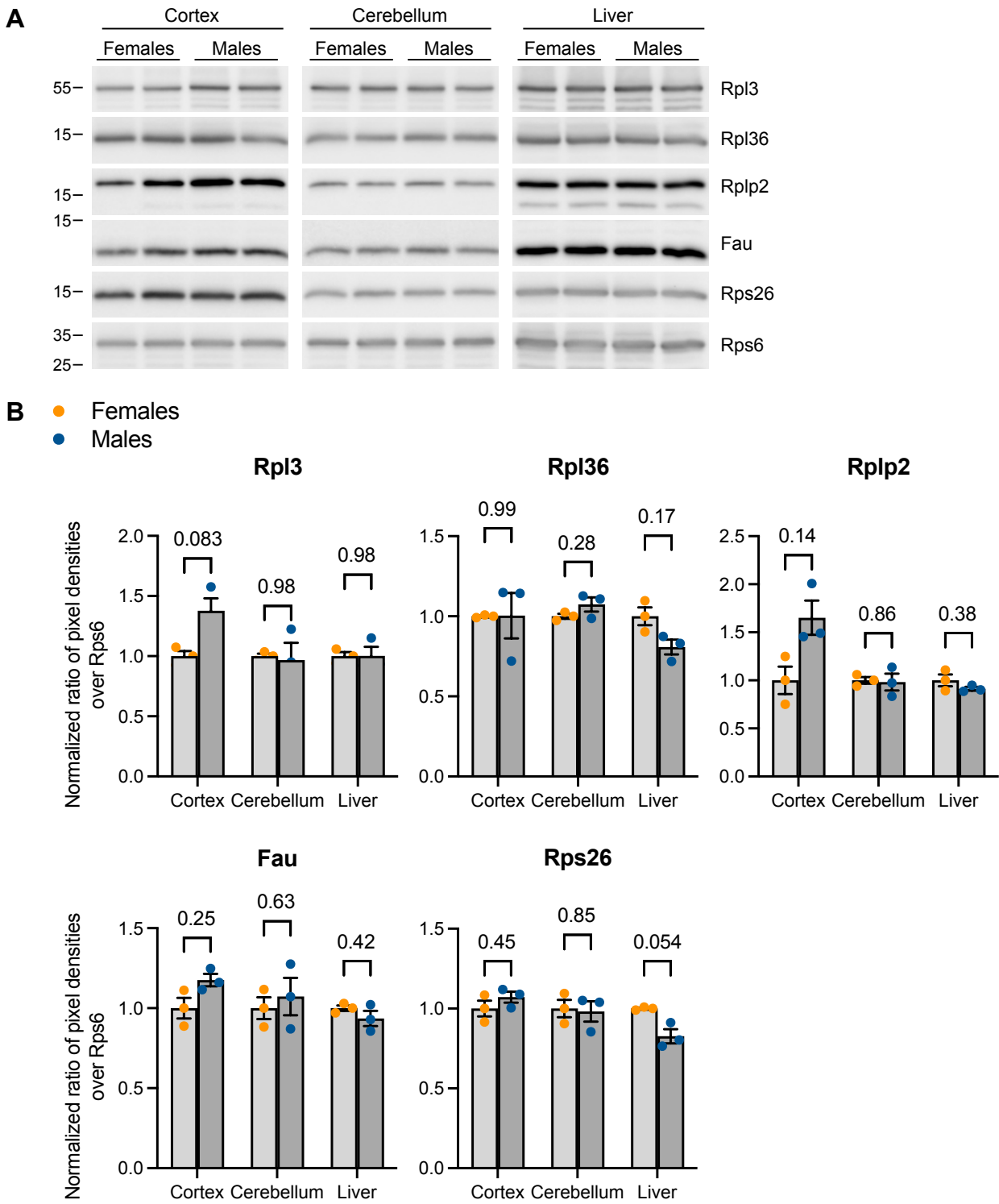

Supplement: Supplementary file 5 — Supplementary Figure 5: Specific RPs show no significant difference in incorporation into ribosomes from females and males. (A) Western blot analysis of the ribosomal fractions obtained from the cortex, the cerebellum and the liver, comparing samples from males versus females: Rpl3, Rpl36, Rplp2, Fau, Rps26 and Rps6. Each well represents one biological replicate. (B) Barplot representation of the pixel density ratio of the RP of interest over Rps6, normalized to the average of female samples. For each RP and each tissue/organ, N=3 biological replicates. For each RP, multiple unpaired t-tests with FDR correction (two-stage step-up method of Benjamini, Krieger and Yekutieli) were conducted. FDR-corrected p-values (q-values) are indicated on each plot [file 18_2025_5708_MOESM5_ESM.pdf]

Supplementary Figure 6

A Rpl10l/Rpl10

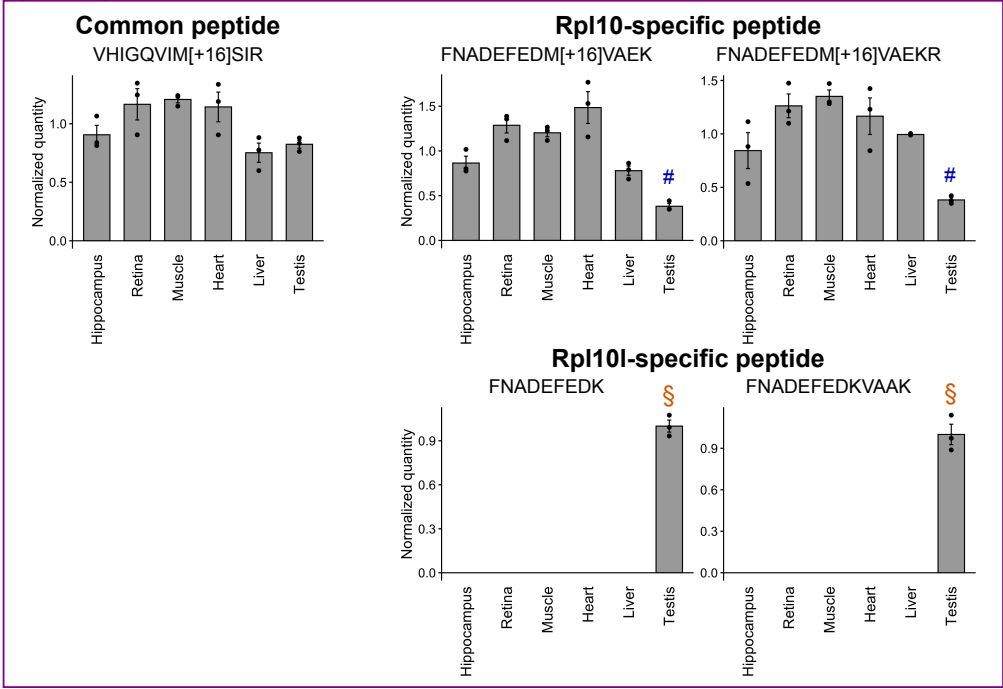

B Rpl3l/Rpl3

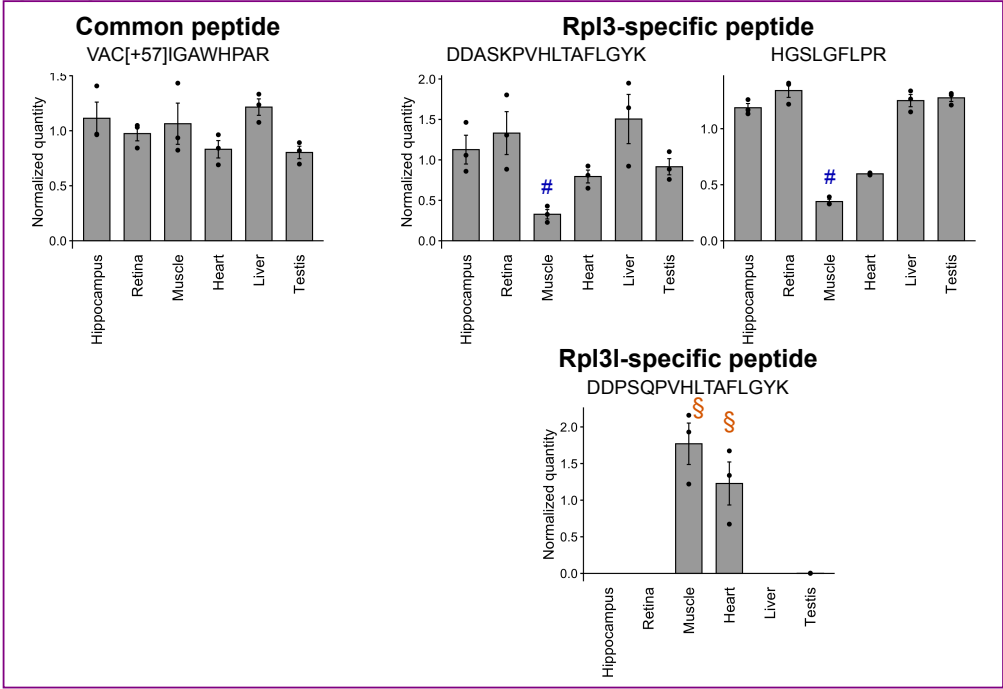

C

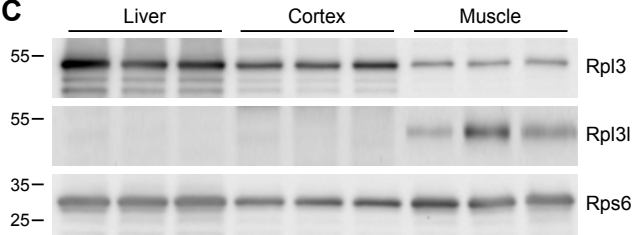

D

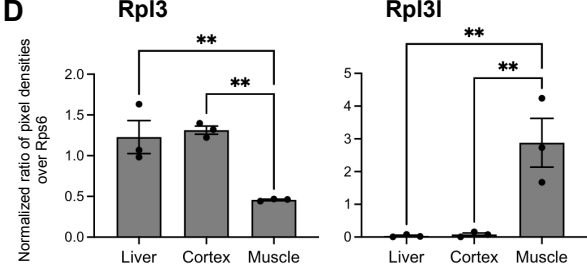

Supplement: Supplementary file 6 — Supplementary Figure 6: Targeted proteomics validates differential abundance of corresponding paralogous and canonical RPs in ribosomal fractions of adult mouse tissues. (A) Barplot representation of the relative abundance in selected tissues of peptides shared by or specific to paralogous Rpl10l and to its corresponding canonical form Rpl10. (B) Barplot representation of the relative abundance in selected tissues of peptides shared by specific to paralogous Rpl3l and to its corresponding canonical form Rpl3. The mean +/- s.e.m. is plotted for each organ, as well as values of individual replicates. # q-value < 0.01 (LIMMA test) and log2FC < -1 for One versus All comparisons. § specific detection. (C) Western blot analysis of the ribosomal fractions obtained in the liver, the cortex and the muscle: Rpl3, Rpl3l, Rps6. Each well represents one biological replicate. (D) Barplot representation of the pixel density ratio of Rpl3 and Rpl3l over Rps6, normalized to the average of all samples. For each tissue/organ, N=3 biological replicates. One-way ANOVA test with Tukey correction for multiple comparisons, ** p-value<0.01 [file 18_2025_5708_MOESM6_ESM.pdf]

Supplementary Figure 7

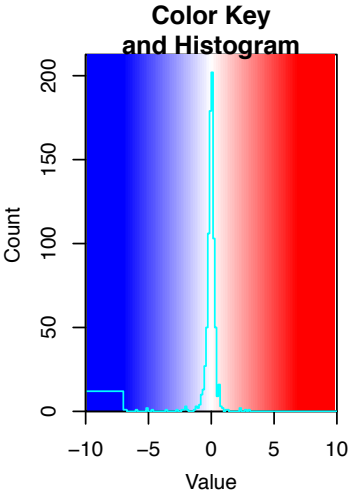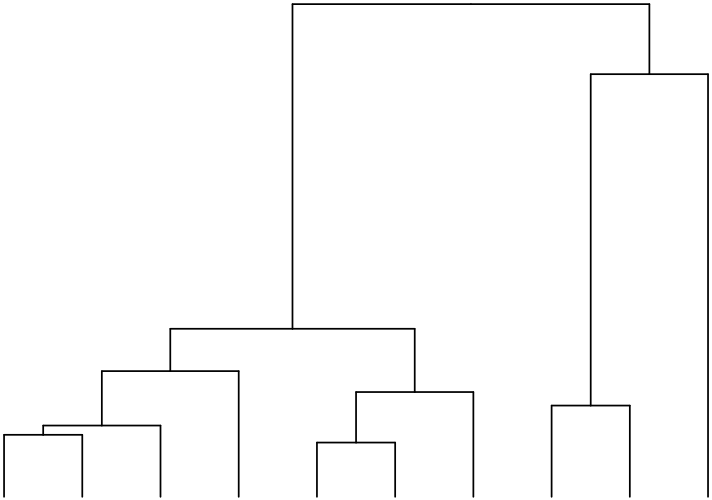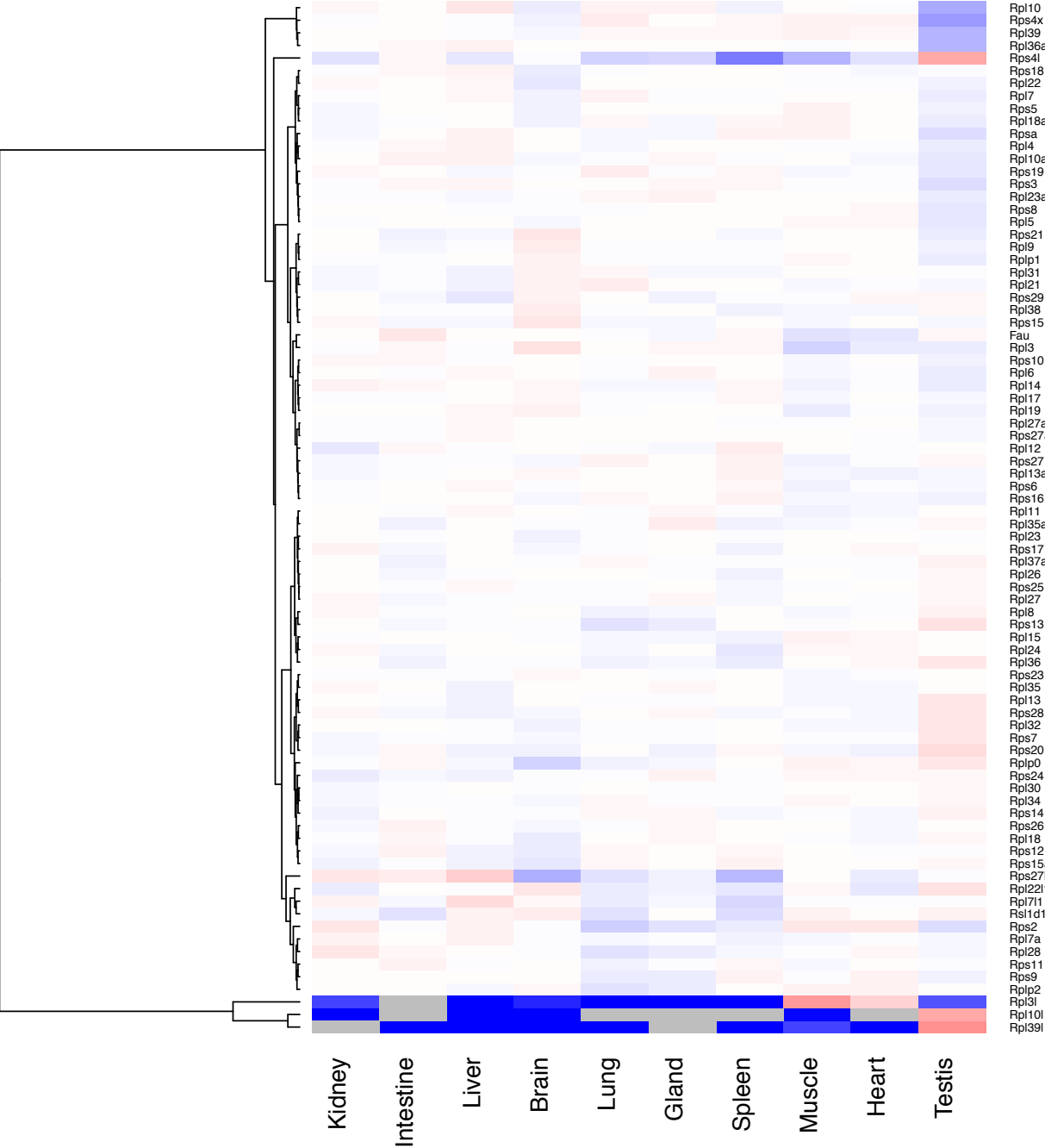

Supplement: Supplementary file 7 — Supplementary Figure 7: Relative expression of RP transcripts across adult mouse tissues. Heatmap of the log-transformed relative expression of RPs detected by RNA-sequencing, from depleted in blue to enriched in red. Grey boxes represent no detection of the RP transcript in the corresponding organ. Expression values from the Mouse Transcriptomic Body Map dataset (58) [file 18_2025_5708_MOESM7_ESM.pdf]
